# Association of TyG index and obesity indicators with cognitive function: a cross - sectional study from Chinese health check-up centers

**DOI:** 10.1186/s12902-026-02280-4

**Published:** 2026-04-17

**Authors:** Jiapei Wei, Xucheng Wu, Liantian Chen, Yincun Wang, Yanjie Zhao, Liming Zhang, Xingqi Cao, Liying Chen, Xuan Ge, Yangzhen Lu, Zuyun Liu, Hui Shentu

**Affiliations:** 1 Department of General Practice, Dongyang People’s Hospital, 60 Wuning West Road, Dongyang, Zhejiang 322100 China; 2 Center for Clinical Big Data and Analytics of the Second Affiliated Hospital and Department of Big Data in Health Science School of Public Health, Zhejiang Key Laboratory of Intelligent Preventive Medicine, Zhejiang University School of Medicine, Hangzhou, 310058 China; 3 Department of General Practice, Sir Run Run Shaw Hospital, Zhejiang University School of Medicine, Hangzhou, Zhejiang 310016 China; 4 Health Management Center, Dongyang People’s Hospital, 60 Wuning West Road, Dongyang, 322100 China

**Keywords:** Triglyceride-glucose index, Cognitive function, Obesity, Metabolism, Early risk detection

## Abstract

**Background:**

Whether the triglyceride-glucose index (TyG) and its derived indices [TyG-body mass index (TyG-BMI), TyG-waist circumference (TyG-WC), TyG-waist-to-height ratio (TyG-WHtR), TyG-weight-adjusted waist index (TyG-WWI), TyG-a body shape index (TyG-ABSI)] are associated with cognitive function remains unclear, particularly in middle-aged populations.

**Methods:**

A total of 876 participants (mean age 49.4 ± 13.3 years) were recruited. The TyG and its related indices were computed and divided into quartiles (Q1-Q4). Cognitive function was assessed using the Montreal Cognitive Assessment (MoCA), Digit Symbol Substitution Test (DSST), and Auditory Verbal Learning Test (AVLT, including immediate recall [AVLT-3] and delayed recall [AVLT-5]). Multivariable linear regression models adjusted for demographic, lifestyle, and clinical covariates (Model 2) were used to assess associations. Restricted cubic spline (RCS) models were applied to explore non-linear relationships.

**Result:**

Higher TyG index quartiles were generally associated with lower cognitive scores, although associations were attenuated compared to models adjusted for age and sex only. Specifically, participants in the highest quartile (Q4) of TyG-WHtR had a 0.97 point lower MoCA score (95% CI: -1.89, -0.06) and a 1.53 point lower DSST score (95% CI: -2.97, -0.10) compared to Q1. Among the TyG-obesity indices, TyG-WHtR and TyG-WC showed consistent and significant associations with MoCA and DSST scores, while TyG-WWI and TyG-ABSI showed weaker or non-significant associations across cognitive domains. These associations were generally more pronounced in females and participants aged < 60 years. RCS analyses indicated approximately linear inverse associations for most indices, with a threshold effect observed for TyG-WWI.

**Conclusions:**

Elevated TyG and its obesity-related indices, particularly TyG-WHtR, are associated with poorer cognitive function in healthy middle-aged Chinese adults. These findings highlight the potential utility of integrating metabolic and anthropometric indicators into early risk assessment for cognitive decline.

**Clinical trial number:**

Not applicable.

**Supplementary Information:**

The online version contains supplementary material available at 10.1186/s12902-026-02280-4.

## Introduction

Cognitive impairment, a neurological disorder marked by deficits in reasoning, memory, and attention, frequently progresses to dementia and is associated with increased mortality [1]. In China, the prevalence of cognitive impairment among adults aged 60 years or older is estimated at approximately 15.5% [2]. Against the backdrop of rapid population aging, cognitive impairment has emerged as an increasingly critical public health concern [3]. Given the substantial personal and social burdens it imposes, identifying modifiable factors associated with cognitive decline is essential for early screening and the development of strategies aimed at preserving cognitive function.

Among the factors consistently associated with cognitive impairment in prospective cohort studies, inflammation and obesity have garnered increasing attention due to their association with metabolic dysfunction, which can be assessed using clinical biomarkers. These include the triglyceride-glucose (TyG) index, a marker of insulin resistance (IR) derived from fasting plasma triglycerides and glucose levels, and several obesity-related indices [4]. Accumulating evidence from prospective studies suggests that a higher TyG index is associated with an elevated risk of cognitive impairment, particularly in older adults [5–9]. However, this association remains underinvestigated in middle-aged populations, who exhibit high prevalence rates of obesity and insulin resistance. Examining the relationship between the TyG index and cognitive function in midlife may reveal preclinical, insulin resistance-related alterations in cognition, thereby offering a valuable opportunity for early intervention. Furthermore, integrating the TyG index with anthropometric indicators such as body mass index (BMI), waist circumference (WC), waist-to-height ratio (WHtR), and a body shape index (ABSI) may more effectively capture the interplay between insulin resistance and cognitive health [10–15]. Such a comprehensive approach may yield novel insights for the prevention of cognitive decline.

In this study, we utilized data from the Zhejiang Health Aging Longitudinal Study (JASHA) to investigate the associations between the TyG index, related obesity indices, and cognitive function in middle-aged adults, with the aim of facilitating earlier identifying of at-risk individuals and informing preventing strategies for cognitive impairment.

## Methods

### Study population

The JASHA study is an ongoing prospective longitudinal cohort study initiated in 2022 at the Health Management Center of Dongyang People’s Hospital, Zhejiang Province, China. Briefly, a total of 876 adults aged ≥ 18 years who underwent annual health examinations were recruited. Data on demographic characteristics, lifestyle factors, and multidimensional health status (including cognitive function) were collected through standardized, face-to-face interviews conducted by trained personnel. The JASHA study protocol was approved by the Ethics Committees of the School of Public Health at Zhejiang University (No. ZGL20212-6) and Dongyang People’s Hospital (No. 2021-YX-206). Written informed consent was obtained from all participants. Further details on the cohort design are available elsewhere.

**Fig. 1:**
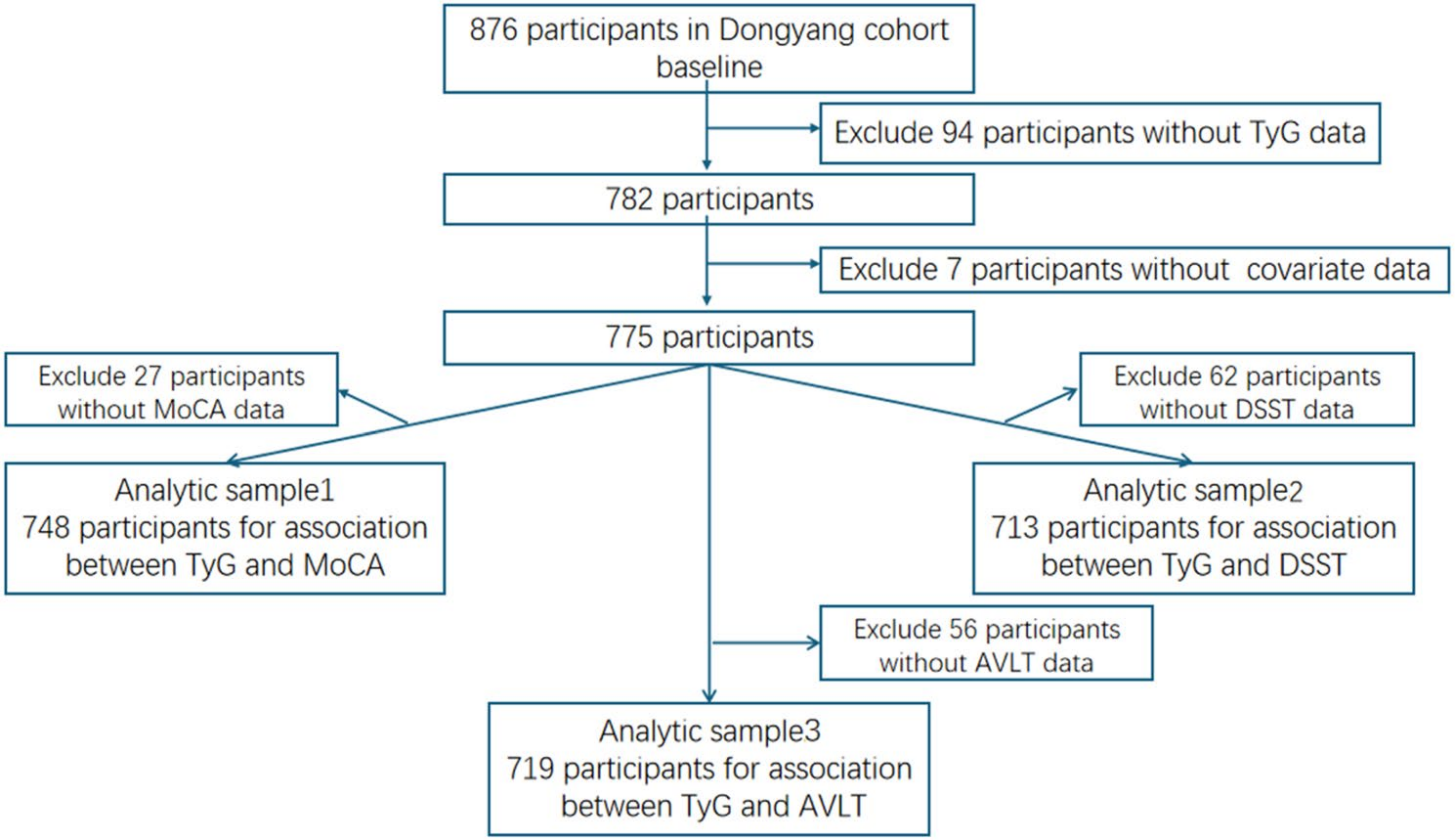
Study flow chart. MoCA, Montreal Cognitive Assessment; AVLT, Auditory Verbal Learning Test DSST, Digit Symbol Substitution Test; TyG, triglyceride-glucose index

For the present analysis, baseline data from 2022 were used. As illustrated in Fig. 1, participants with complete data on the TyG index and covariates were initially included (*n* = 775). Subsequently, three overlapping analytical samples were defined based on the availability of cognitive test data: 1) Sample 1 included participants with complete Montreal Cognitive Assessment (MoCA) data (*n* = 748); 2) Sample 2 included those with complete Digit Symbol Substitution Test (DSST) data (*n* = 713); and 3) Sample 3 included those with complete Auditory Verbal Learning Test (AVLT) data (*n* = 719). This analytical strategy was used to maximize the sample size for each cognitive outcome, rather than restricting the analysis to participants with complete data for all three cognitive tests, which would reduce statistical power and introduce additional selection bias.”

## Assessment of TyG index and related obesity indices

### Biochemical analysis

Overnight fasting blood samples were collected by trained nurses using heparin-anticoagulated vacuum tubes and promptly transported to the central hospital laboratory. Plasma was separated by centrifugation and stored at -80 °C ​until analysis. Plasma triglyceride (TG) and fasting plasma glucose (FPG) levels were measured using standardized automated assays. Detailed descriptions of laboratory procedures are provided elsewhere.

### Anthropometric measurements and obesity indices

Trained healthcare professionals recorded body weight, height, and waist circumference (WC) during physical examinations. These measurements were used to calculate the following obesity-related indices:

(1) $$\:\mathrm{B}\mathrm{M}\mathrm{I}=\frac{\mathrm{W}\mathrm{e}\mathrm{i}\mathrm{g}\mathrm{h}\mathrm{t}\:\left(\mathrm{k}\mathrm{g}\right)\text{}}{{\mathrm{H}\mathrm{e}\mathrm{i}\mathrm{g}\mathrm{h}\mathrm{t}\:\left(\mathrm{m}\right)}^{2}}$$

(2) $$\:\mathrm{W}\mathrm{H}\mathrm{t}\mathrm{R}=\frac{\mathrm{W}\mathrm{C}\:\left(\mathrm{c}\mathrm{m}\right)\text{}}{\mathrm{H}\mathrm{e}\mathrm{i}\mathrm{g}\mathrm{h}\mathrm{t}\:\left(\mathrm{c}\mathrm{m}\right)}$$

(3) $$\:\mathrm{W}\mathrm{W}\mathrm{I}=\frac{\mathrm{W}\mathrm{C}\:\left(\mathrm{c}\mathrm{m}\right)\text{}}{\sqrt{\mathrm{W}\mathrm{e}\mathrm{i}\mathrm{g}\mathrm{h}\mathrm{t}\:\left(\mathrm{k}\mathrm{g}\right)}}$$

(4) $$\:\mathrm{A}\mathrm{B}\mathrm{S}\mathrm{I}=\frac{\mathrm{W}\mathrm{C}\:\left(\mathrm{m}\right)\text{}}{{\mathrm{B}\mathrm{M}\mathrm{I}}^{2/3}\times\:{\mathrm{H}\mathrm{e}\mathrm{i}\mathrm{g}\mathrm{h}\mathrm{t}}^{1/2}}$$

### TyG index and related obesity indices

The TyG index was calculated as: (Both TG and FPG were measured in mg/dL for this calculation to ensure reproducibility).

$$\:\mathrm{T}\mathrm{y}\mathrm{G}\:\mathrm{i}\mathrm{n}\mathrm{d}\mathrm{e}\mathrm{x}=\mathrm{ln}\:[\frac{\mathrm{T}\mathrm{G}\:\left(\frac{\mathrm{m}\mathrm{g}}{\mathrm{d}\mathrm{L}}\right)\times\:\mathrm{F}\mathrm{P}\mathrm{G}\:\left(\frac{\mathrm{m}\mathrm{g}}{\mathrm{d}\mathrm{L}}\right)}{2}]$$

Five composite metabolic-obesity indices were then derived by multiplying the TyG index by each of the above anthropometric measures [10, 15, 16]:

1. TyG-BMI = TyG index × BMI
2. TyG-WC = TyG index × WC (cm)
3. TyG-WHtR = TyG index × WHtR
4. TyG-WWI = TyG index × WWI
5. TyG-ABSI = TyG index × ABSI

Participants were subsequently stratified into four quartiles (Q1-Q4) for each index based on the distribution of the TyG index and the composite indices.

### Assessment of cognitive function

Cognitive function was evaluated using three validated neuropsychological tests: the MoCA, the DSST, and the AVLT. All cognitive assessments were conducted by trained research personnel who completed a standardized training program on the test protocols, to ensure consistency of administration.

### MoCA

The Chinese version of the MoCA, a 30 point screening tool, was used to assess five cognitive domains: executive function, memory, language, attention, and orientation [17]. Total scores range from 0 to 30, with higher scores indicating better cognitive performance.

### DSST

The DSST was administered to evaluate processing speed and sustained attention [18]. Participants matched symbols to numbers (1–9) according to a standard key within 90 s. The score reflects the total number of correct matches, with higher values indicating better cognitive function [19].

### AVLT

The AVLT was used to assess episodic memory function [19–21], yielding two primary outcomes: immediate recall score from the third trial (AVLT-3, score range: 0–36) and delayed recall ( AVLT-5, score range: 0–60). Higher scores indicate better memory performance.

### Covariates

The following covariates were included in this study: demographic characteristics (age [continuous], gender [male/female], educational level [primary school or uneducated/junior or senior high school/college and above]), Medical history (self-reported hypertension), and lifestyle factors (physical activity [measured in metabolic equivalent of task minutes per week, MET-min/week], smoking status[current/ former/never], and alcohol consumption [current/ former/never]).

Physical activity was assessed using the validated short form of the International Physical Activity Questionnaire (IPAQ) [22]. Weekly MET-min were calculated based on reported activity in the prior week and categorized as low, moderate, or high [23, 24]. Given its potential confounding effect, low physical activity was also analyzed as a binary variable (low = yes, moderate/high = no).

### Statistical analyses

#### Descriptive analyses

Participant characteristics were summarized for the overall sample and stratified by quartiles of the TyG index. Continuous variables were expressed as mean ± standard deviation (SD), and categorical variables as counts (percentages). Differences across quartiles were assessed using one-way ANOVA for continuous variables and chi-square tests for categorical variables. Distributions of cognitive scores (MoCA, DSST, AVLT-3, AVLT-5) were evaluated for skewness, kurtosis, and potential ceiling effects.

#### Primary associational analyses

Associations between the TyG index, TyG-based obesity indices (TyG-BMI, TyG-WC, TyG-WHtR, TyG-WWI, TyG-ABSI), and cognitive outcomes were examined using multivariable linear regression models. Each index was analyzed as a continuous variable, and both the original-unit β and standardized β (per 1-SD increase) were reported. Two models were applied: Model 1 included age and sex; Model 2 additionally included education level, smoking status, alcohol consumption, physical activity, hypertension, and total cholesterol. To avoid over-adjustment, in models for composite indices, the corresponding anthropometric component was excluded from covariates (e.g., BMI was not adjusted in the TyG-BMI model; WC was not adjusted in the TyG-WC, TyG-WHtR, TyG-WWI, and TyG-ABSI models). Variance inflation factors (VIF) were computed to ensure collinearity among exposure and covariates was within acceptable limits.

Restricted cubic spline (RCS) models with three knots at the 10th, 50th, and 90th percentiles of the exposure distribution were applied to examine non-linear relationships. Reference values were set at the 10th percentile. For indices with significant non-linearity (e.g., TyG-WWI), segmented linear regression was conducted to estimate inflection points and effect sizes before and after the threshold. Rug plots were included to visualize data density.

### Additional analyses

#### Stratified analyses and effect modification

Analyses were stratified by sex, age (< 60 vs. ≥60 years) and BMI(< 24 vs. ≥24). Formal interaction tests (TyG × sex; TyG × age group; TyG × BMI category) were performed. Subgroup sample sizes were reported.

#### Sensitivity analyses

Analyses were repeated excluding participants with diabetes, cardiovascular disease, stroke, or use of glucose-/lipid-lowering medications. Age-stratified analyses were further conducted using median and quartile cutoffs. Multiple imputation using chained equations (MICE) was applied for missing data, and results were compared with complete-case analyses.

#### Independent and joint effects

Three parallel models were fitted to assess independent and interactive contributions: Model A included the TyG index and covariates; Model B included the obesity index and covariates; Model C included both the TyG and obesity indices as independent variables.

#### Clinical effect quantification

Cognitive impairment was defined as MoCA < 26. Logistic regression was used to estimate odds ratios (ORs) and 95% confidence intervals (CIs) for cognitive impairment per unit increase in each index.

#### Model fit and predictive value comparison

For each cognitive outcome, model fit was evaluated using R², Akaike Information Criterion (AIC), and Bayesian Information Criterion (BIC). Comparisons were made between models including only TyG, only obesity indices, or TyG-obesity composite indices to assess incremental predictive value.

All analyses were conducted using R 4.4.2. Two-sided *P* < 0.05 was considered statistically significant unless FDR-adjusted P values indicated otherwise.

## Results

### Basic characteristics of study participants

The mean (SD) age of participants included in the MoCA analysis was 49.4 (13.3) years, and 45.2% (*n* = 338) were female (Table 1). Participants with higher TyG levels tended to be older, more likely male, and more likely to consume alcohol, and they had higher BMI, total cholesterol, and prevalence of hypertension (all *P* < 0.05). Higher TyG levels were also associated with lower MoCA scores and higher values of TyG-related obesity indices. Similar baseline characteristics were observed in the DSST (*N* = 713) and AVLT (*N* = 719) samples (Supplementary Tables S1–S2).

**Table 1:** Baseline characteristics of the study population (MoCA)

Characteristics	Overall (*N* = 748)	Triglyceride-glucose index				*P*
		Quartile 1 (*N* = 188)	Quartile 2 (*N* = 186)	Quartile 3 (*N* = 185)	Quartile 4 (*N* = 189)	
Age, years (mean (SD))	49.4 (13.3)	45.3 (14.1)	50.7 (13.7)	50.8 (13.2)	51.0 (11.4)	**< 0.001**
gender, female, n (%)	338 (45.2)	120 (63.8)	96 (51.6)	75 (40.5)	47 (24.9)	**< 0.001**
Education level, n (%)						0.102
Less than primary school	63 (8.4)	12 (6.4)	16 (8.6)	19 (10.3)	16 (8.5)	
High school or equivalent	315 (42.1)	67 (35.6)	78 (41.9)	77 (41.6)	93 (49.2)	
College or above	370 (49.5)	109 (58.0)	92 (49.5)	89 (48.1)	80 (42.3)	
Alcohol = yes, n (%)	311 (41.6)	50 (26.6)	70 (37.6)	87 (47.0)	104 (55.0)	**< 0.001**
Smoking = yes, n (%)	204 (27.3)	29 (15.4)	41 (22.0)	58 (31.4)	76 (40.2)	**< 0.001**
BMI (mean (SD))	23.9 (3.5)	21.5 (2.9)	23.3 (3.1)	25.0 (3.3)	25.8 (3.0)	**< 0.001**
Activity = low, n (%)	87 (11.6)	20 (10.6)	21 (11.3)	20 (10.8)	26 (13.8)	0.764
TC (mean (SD))	4.9 (1.0)	4.6 (0.9)	4.8 (0.9)	4.9 (0.9)	5.2 (1.0)	**< 0.001**
Hypertension = Yes, n (%)	145 (19.4)	11 (5.9)	29 (15.6)	41 (22.2)	64 (33.9)	**< 0.001**
MoCA (mean (SD))	25.2 (3.8)	26.0 (3.1)	25.2 (4.2)	25.0 (3.5)	24.7 (4.1)	**0.007**
TyG-BMI (mean (SD))	206.8(40.3)	167.7 (24.0)	194.1 (25.2)	218.7 (29.5)	246.3 (31.5)	**< 0.001**
TyG-WC (mean (SD))	735.2 (132.8)	602.5 (71.7)	693.1 (78.1)	770.4(96.6)	874.2 (99.0)	**< 0.001**
TyG-WHtR (mean (SD))	4.4 (0.8)	3.7 (0.4)	4.2 (0.5)	4.7 (0.52)	5.2 (0.5)	**< 0.001**
TyG-WWI (mean (SD))	90.9 (11.0)	79.3 (5.5)	87.5 (6.3)	93.4 (6.8)	103.2 (7.8)	**< 0.001**
TyG-ABSI (mean (SD))	6.9 (0.8)	6.1(0.4)	6.6 (0.4)	7.0 (0.5)	7.8 (0.6)	**< 0.001**

Note: SD, standard deviation; TC, total cholesterol; MoCA, Montreal Cognitive Assessment; TyG, triglyceride-glucose index; WHtR, waist-to-height ratio; BMI, body mass index; WC, waist circumference; WWI, weight-adjusted waist index; ABSI, a body shape index

Continuous variables were presented as mean (SD); categorical variables were presented as numbers (percentages). Group differences were compared using Analysis of Variance (ANOVA) and the chi-square test, respectively. Percentages may not total 100 due to rounding

### Association of TyG index and related obesity indices with cognitive function

**Fig. 2:**
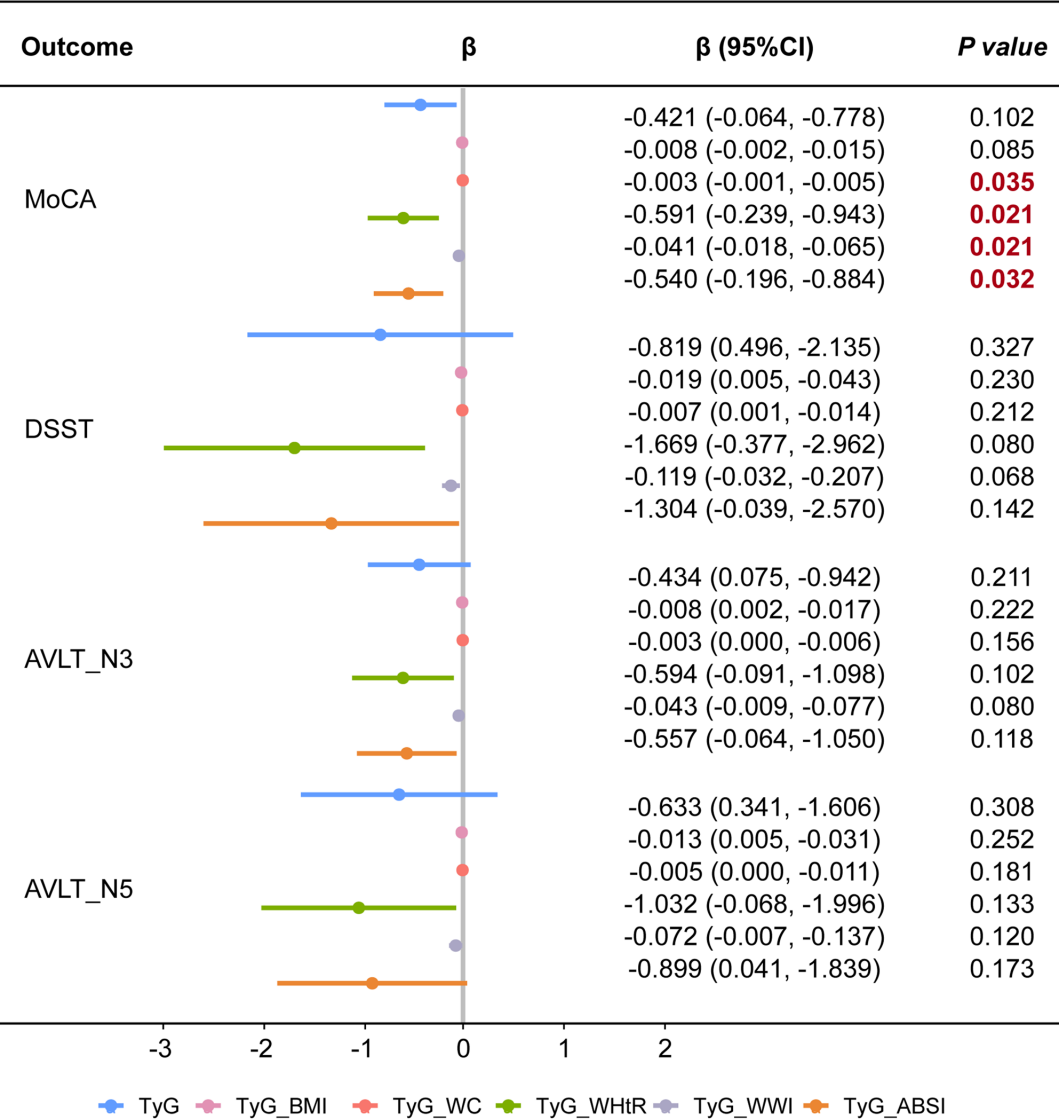
Association between TyG and related obesity indices with cognitive function. Notes: CI, confidence intervals, MoCA, Montreal Cognitive Assessment; DSST, Digit Symbol Substitution Test; AVLT-3, Auditory Verbal Learning Test-Immediate Recall Trial 3; AVLT-5, Auditory Verbal Learning Test-Delayed Recall; CI, confidence interval; TyG, triglyceride-glucose index; WHtR, waist-to-height ratio; BMI, body mass index; WC, waist circumference; WWI, weight-adjusted waist index; ABSI, a body shape index. The 95% Cls are unadjusted; p-values are FDR adjusted using the Benjamini-Hochberg procedure. Adjusted for gender and age

Figure 2 shows the associations between the TyG index and TyG-based obesity indices (TyG-BMI, TyG-WC, TyG-WHtR, TyG-WWI, TyG-ABSI) and cognitive function in Model 1 (adjusted for age and sex) using original-unit β. For MoCA, TyG-WC, TyG-WHtR, TyG-WWI, and TyG-ABSI were significantly associated with scores, with β coefficients ranging from − 0.591 to -0.003 (all *P* < 0.05).

For DSST, higher TyG-related indices were generally associated with lower cognitive performance, although most associations did not reach statistical significance. Similarly, for AVLT outcomes (AVLT-3 and AVLT-5), higher TyG and TyG-based obesity indices were consistently associated with lower memory scores, but these associations were modest and not statistically significant.

Standardized β estimates for both Model 1 and Model 2 are presented in Supplementary Table S4. Overall, the direction of associations remained consistent across outcomes, with higher TyG-related indices associated with poorer cognitive performance, although effect sizes were attenuated after full adjustment.

Table 2 present the associations between the TyG index quartiles and cognitive performance. After adjustment for gender and age (Model 1), a higher TyG index was significantly associated with poorer cognitive function. Specifically, participants in the highest quartile (Q4) had a 0.81point lower MoCA score compared to those in Q1 (95% CI: -1.51, -0.10). Additionally, per 1-SD increase in the TyG index, MoCA scores decreased by 0.42 points (95% CI: -0.78, -0.06). Among the TyG-obesity indices, TyG-WHtR and TyG-WWI showed consistent and significant associations with all cognitive function outcome. For example, a 1-SD increase in TyG-WHtR was associated with a 0.59 point lower MoCA score (95% CI: -1.94, -0.24), a 1.67point lower DSST score (95% CI: -2.96, -0.38), a 0.59 point lower AVLT-3 score (95% CI: -1.10, -0.09) and a 1.03 point lower AVLT-5 score (95% CI: -2.00, -0.07).

**Table 2:** Association of TyG and its composite indices quartiles with cognitive function (Model1)

	MOCA Beta (95%CI)	DSST Beta (95%CI)	AVLT-N3 Beta (95%CI)	AVLT-N5 Beta (95%CI)
TyG				
Quartile 1	**Ref.**			
Quartile 2	-0.43 (-1.11, 0.26)	-0.85 (-3.35, 1.66)	-0.27 (-1.25, 0.70)	-0.23 (-2.12, 1.65)
Quartile 3	-0.31 (-1.00, 0.39)	-0.66 (-3.19, 1.87)	-0.6 (-1.59, 0.39)	-0.86 (-2.76, 1.03)
Quartile 4	**-0.81 (-1.51**, -0.10) *	-1.95 (-4.55, 0.64)	-0.85 (-1.86, 0.16)	-1.16 (-3.10, 0.78)
TyG-BMI				
Quartile 1	**Ref.**			
Quartile 2	-0.25 (-0.93, 0.44)	0.35 (-2.15, 2.85)	-0.53 (-1.51, 0.45)	-0.13 (-2.01, 1.76)
Quartile 3	-0.65 (-1.35, 0.06)	-0.96 (-3.56, 1.63)	-0.84 (-1.84, 0.17)	-1.15 (-3.08, 0.79)
Quartile 4	**-0.87 (-1.60**, -0.14)*	-2.10 (-4.79, 0.58)	-0.76 (-1.80, 0.29)	-1.02 (-3.03, 1.00)
TyG-WC				
Quartile 1	**Ref.**			
Quartile 2	0.01 (-0.67, 0.70)	-0.06 (-2.58, 2.47)	0.31 (-0.67, 1.30)	0.61 (-1.29, 2.50)
Quartile 3	**-0.74 (-1.48**, -0.01)*	-2.22 (-4.93, 0.50)	-0.33 (-1.38, 0.73)	-0.07 (-2.10, 1.97)
Quartile 4	**-0.99 (-1.77**, -0.21)*	-1.51 (-4.39, 1.37)	-0.69 (-1.81, 0.43)	-1.23 (-3.38, 0.92)
TyG-WHtR				
Quartile 1	**Ref.**			
Quartile 2	-0.24 (-0.91, 0.44)	-1.34 (-3.83, 1.15)	-0.36 (-1.33, 0.61)	-0.32 (-2.19, 1.55)
Quartile 3	0.04 (-0.66, 0.73)	-1.96 (-4.54, 0.61)	-0.13 (-1.14, 0.87)	0.34 (-1.58, 2.26)
Quartile 4	**-1.35 (-2.07**, -0.63)***	**-3.70 (-6.36**, -1.04)**	**-1.47 (-2.5**, -0.44)**	**-2.46 (-4.44**, -0.48)*
TyG-WWI				
Quartile 1	**Ref.**			
Quartile 2	0.26 (-0.41, 0.94)	-0.48 (-2.96, 2.01)	-0.23 (-1.19, 0.74)	0.17 (-1.70, 2.04)
Quartile 3	0.05 (-0.65, 0.76)	-1.89 (-4.48, 0.7)	-0.02 (-1.03, 0.99)	0.64 (-1.30, 2.58)
Quartile 4	**-1.06 (-1.78**, -0.33)**	**-2.9 (-5.57**, -0.22)*	**-1.17 (-2.21**, -0.13)*	-1.71 (-3.70, 0.28)
TyG-ABSI				
Quartile 1	**Ref.**			
Quartile 2	0.06 (-0.63, 0.74)	-1.18 (-3.68, 1.32)	0.33 (-0.65, 1.30)	0.59 (-1.29, 2.47)
Quartile 3	-0.29 (-1.01, 0.43)	-1.94 (-4.60, 0.71)	-0.19 (-1.22, 0.84)	0.31 (-1.68, 2.30)
Quartile 4	**-0.93 (-1.68**, -0.18)*	-2.39 (-5.14, 0.36)	-1.01 (-2.07, 0.06)	-1.69 (-3.73, 0.36)

Notes: MoCA, Montreal Cognitive Assessment; DSST, Digit Symbol Substitution Test; AVLT-3, Auditory Verbal Learning Test-Immediate Recall Trial 3; AVLT-5, Auditory Verbal Learning Test-Delayed Recall; CI, confidence interval; TyG, triglyceride-glucose index; WHtR, waist-to-height ratio; BMI, body mass index; WC, waist circumference; WWI, weight-adjusted waist index; ABSI, a body shape index

Adjusted for gender and age

* *p* < 0.05; ** *p* < 0.01; *** *p* < 0.001

Table S4 presents the associations between the TyG index quartiles and cognitive performance after multivariable adjustment (Model 2). After adjustment for covariates, higher TyG index quartiles were generally associated with lower cognitive scores, although the associations were attenuated compared to Model 1. Specifically, participants in the highest quartile (Q4) of TyG-WHtR had a 0.97 point lower MoCA score (95% CI: -1.89, -0.06) and a 1.53 point lower DSST score (95% CI: -2.97, -0.10) compared to those in Q1. Among the TyG-obesity indices, TyG-WHtR and TyG-WC showed significant associations with MoCA and DSST scores, while TyG-WWI and TyG-ABSI showed weaker or non-significant associations across cognitive domains. These findings suggest that higher TyG-WHtR remains consistently associated with poorer cognitive function even after extensive covariate adjustment.

**Fig. 3:**
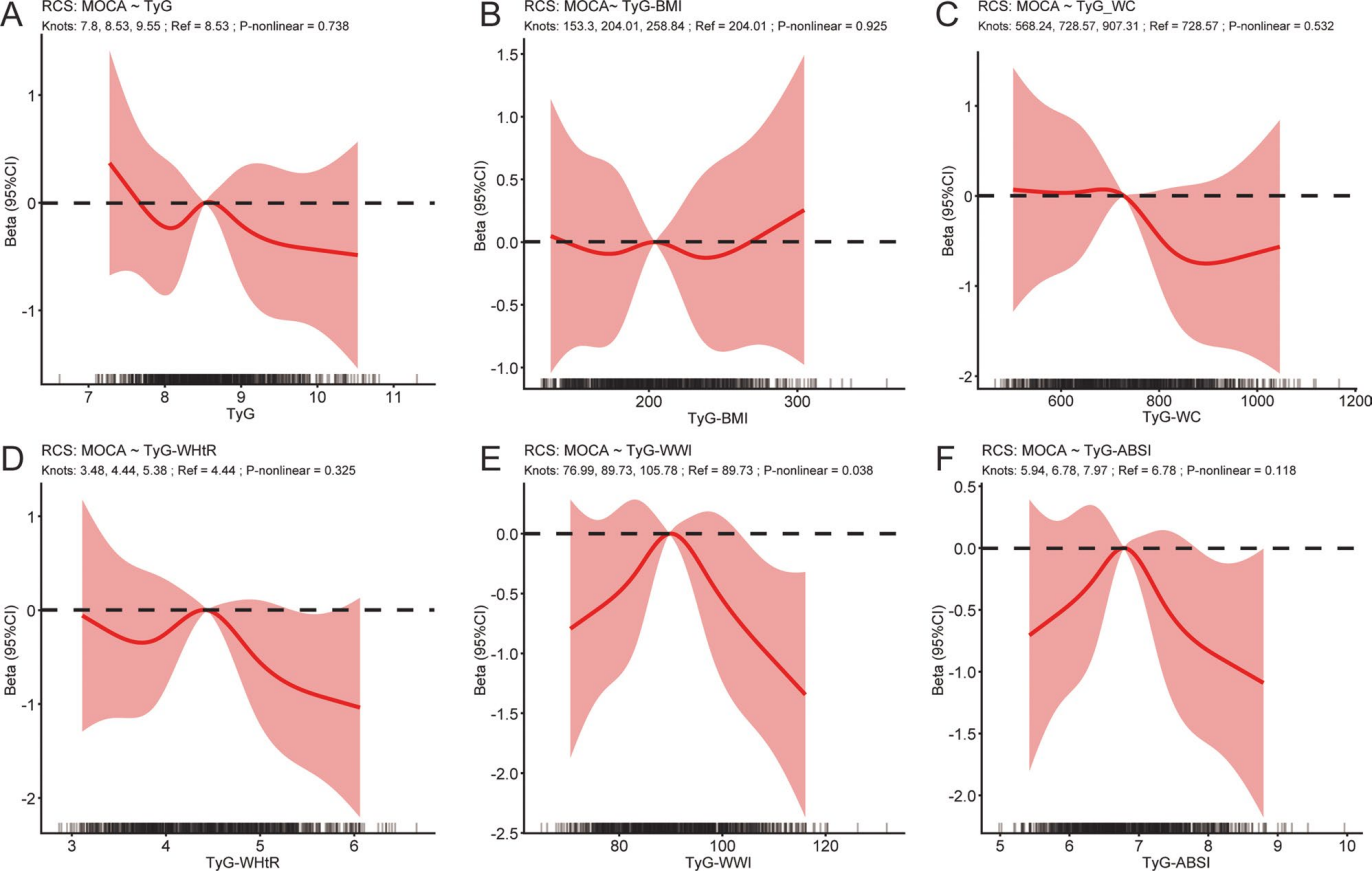
Restricted cubic spline (RCS) analyses of the associations between TyG and related obesity indices with MoCA^a^. **Panels A-F**: RCS curves for TyG, TyG-BMI, TyG-WC, TyG-WHtR, TyG-WWI, and TyG-ABSI and MoCA. a Adjusted for gender, age, education level, alcohol consumption, smoking status, body mass index (BMI), total cholesterol, physical activity, and history of hypertension. To avoid over-adjustment bias, the corresponding anthropometric component was excluded from covariates in models for each composite index

Figure 3 presents the restricted cubic spline (RCS) analyses of the associations between the TyG index and TyG-based obesity indices and MoCA scores.Overall, most indices showed approximately linear inverse associations with MoCA scores, as no significant non-linearity was observed for TyG (P for non-linearity = 0.738), TyG-BMI (P for non-linearity = 0.925), TyG-WC (P for non-linearity = 0.532), TyG-WHtR (P for non-linearity = 0.325), or TyG-ABSI (P for non-linearity = 0.118).In contrast, a significant non-linear association was observed for TyG-WWI (P for non-linearity = 0.038). Specifically, the inverse association between TyG-WWI and MoCA scores was more pronounced at lower levels of TyG-WWI, followed by a plateau at higher values.To further characterize the observed non-linear association, segmented linear regression analysis was performed for TyG-WWI. An inflection point was identified at approximately 89.7. Below this threshold, higher TyG-WWI was associated with a steeper decline in MoCA scores, whereas the association became attenuated and plateaued above this level.

### Additional analysis

Stratified analyses by sex, age (< 60 vs. ≥60 years), and BMI category were performed to assess potential effect modification ( Table 3). Overall, the direction of associations between TyG-related indices and cognitive outcomes was generally consistent across subgroups. Some significant interactions by sex were observed in Model 1; however, these interactions were attenuated and no longer statistically significant after further adjustment in Model 2 (Supplementary Table S5). No significant interactions were observed for age or BMI in either model. In BMI-stratified analyses, some associations appeared more pronounced among non-overweight individuals. For example, TyG-WHtR, TyG-WWI, and TyG-ABSI showed significant inverse associations with AVLT-3 scores in the non-overweight group, whereas these associations were not observed in overweight individuals. Additional analyses using age quartiles yielded comparable results in both Model 1 and Model 2, with no consistent evidence of effect modification across strata (Supplementary Tables S7–S8).

**Table 3:** Subgroup analyses of the association between TyG and related obesity indices with cognitive function (Model1)

Subgroup	Exposure	MoCA Beta (95%CI)	DSST Beta (95%CI)	AVLT-3 Beta (95%CI)	AVLT-5 Beta (95%CI)
**Gender**					
Male	TyG	-0.06 (-0.45, 0.33)	1.00 (-0.51, 2.52)	-0.14 (-0.76, 0.47)	-0.24 (-1.41, 0.94)
	TyG-BMI	-0.00 (-0.01, 0.01)	0.02 (-0.01, 0.05)	-0.00 (-0.01, 0.01)	-0.01 (-0.03, 0.02)
	TyG-WC	-0.00 (-0.00, 0.00)	0.01 (-0.00, 0.02)	-0.00 (-0.00, 0.00)	-0.00 (-0.01, 0.01)
	TyG-WHtR	-0.17 (-0.57, 0.23)	0.57 (-0.96, 2.09)	-0.18 (-0.81, 0.45)	-0.33 (-1.53, 0.87)
	TyG-WWI	-0.01 (-0.04, 0.02)	0.01 (-0.09, 0.12)	-0.01 (-0.05, 0.03)	-0.02 (-0.10, 0.06)
	TyG-ABSI	-0.13 (-0.52, 0.25)	0.37 (-1.10, 1.84)	-0.08 (-0.69, 0.52)	-0.18 (-1.33, 0.96)
Female	TyG	-0.76 (-1.46, -0.05)	-2.27 (-4.72, 0.17)	-0.71 (-1.65, 0.23)	-0.81 (-2.62, 1.00)
	TyG-BMI	**-0.93 (-1.61**, -0.25)*	**-3.45 (-5.80**, -1.11)*	-1.02 (-1.93, -0.12)	-1.68 (-3.41, 0.05)
	TyG-WC	-0.01 (-0.02, 0.00)	-0.05 (-0.09, -0.01)	-0.01 (-0.03, 0.01)	-0.01 (-0.04, 0.02)
	TyG-WHtR	-0.01 (-0.01, -0.00)	-0.02 (-0.03, -0.00)	-0.01 (-0.01, -0.00)	-0.01 (-0.02, 0.00)
	TyG-WWI	**-0.07 (-0.12**, -0.03)*	**-0.22 (-0.38**, -0.06)*	**-0.08 (-0.15**, -0.02)*	-0.13 (-0.25, -0.01)
	TyG-ABSI	**-0.96 (-1.63**, -0.30)*	-2.49 (-4.80, -0.17)	**-1.21 (-2.10**, -0.32)*	-1.76 (-3.47, -0.04)
**Age**					
< 60 Years	TyG	-0.35 (-0.70, 0.01)	-0.77 (-2.24, 0.70)	-0.36 (-0.96, 0.23)	-0.43 (-1.55, 0.69)
	TyG-BMI	**-0.49 (-0.86**, -0.13)*	-1.60 (-3.09, -0.11)	-0.54 (-1.14, 0.07)	-0.81 (-1.96, 0.34)
	TyG-WC	-0.01 (-0.01, 0.00)	-0.02 (-0.04, 0.01)	-0.01 (-0.02, 0.01)	-0.01 (-0.03, 0.01)
	TyG-WHtR	-0.00 (-0.01, 0.00)	-0.01 (-0.02, 0.00)	-0.00 (-0.01, 0.00)	-0.00 (-0.01, 0.00)
	TyG-WWI	**-0.04 (-0.06**, -0.01)*	-0.11 (-0.21, -0.01)	-0.04 (-0.08, 0.00)	-0.06 (-0.14, 0.02)
	TyG-ABSI	**-0.47 (-0.81**, -0.13)*	-1.28 (-2.69, 0.14)	-0.51 (-1.08, 0.06)	-0.74 (-1.82, 0.35)
≥ 60 Years	TyG				
	TyG-BMI	-0.44 (-1.66, 0.77)	2.31 (-0.98, 5.59)	-0.62 (-1.76, 0.52)	-1.26 (-3.53, 1.01)
	TyG-WC	-0.65 (-1.71, 0.41)	0.54 (-2.33, 3.41)	-0.64 (-1.64, 0.37)	-1.53 (-3.53, 0.48)
	TyG-WHtR	-0.01 (-0.03, 0.01)	0.03 (-0.03, 0.08)	-0.01 (-0.03, 0.01)	-0.03 (-0.07, 0.01)
	TyG-WWI	-0.00 (-0.01, 0.00)	0.01 (-0.01, 0.03)	-0.00 (-0.01, 0.00)	-0.01 (-0.02, 0.00)
	TyG-ABSI	-0.05 (-0.12, 0.03)	0.02 (-0.18, 0.22)	-0.04 (-0.11, 0.03)	-0.09 (-0.23, 0.05)
**BMI**					
Non overweight	TyG	-0.49 (-1.02, 0.04)	-0.25 (-2.35, 1.85)	-0.74 (-1.49, 0.02)	-0.71 (-2.17, 0.75)
	TyG-BMI	-0.69 (-1.33, -0.05)	-1.01 (-3.56, 1.54)	-1.18 (-2.09, -0.28)	-1.78 (-3.53, -0.02)
	TyG-WC	-0.01 (-0.02, 0.01)	0.00 (-0.05, 0.06)	-0.02 (-0.04, 0.00)	-0.02 (-0.06, 0.02)
	TyG-WHtR	-0.00 (-0.01, 0.00)	-0.00 (-0.02, 0.01)	-0.01 (-0.01, 0.00)	-0.01 (-0.02, 0.00)
	TyG-WWI	**-0.05 (-0.08**, -0.01)*	-0.08 (-0.22, 0.06)	**-0.07 (-0.12**, -0.02)*	-0.10 (-0.20, 0.00)
	TyG-ABSI	**-0.60 (-1.10**, -0.11)*	-0.97 (-2.92, 0.99)	**-0.94 (-1.64**, -0.24)*	-1.23 (-2.58, 0.12)
Overweight	TyG	-0.31 (-0.85, 0.24)	-0.98 (-2.80, 0.85)	-0.25 (-1.03, 0.53)	-0.73 (-2.19, 0.74)
	TyG-BMI	-0.73 (-1.37, -0.09)	-2.49 (-4.62, -0.35)	-0.64 (-1.57, 0.28)	-1.44 (-3.18, 0.31)
	TyG-WC	-0.01 (-0.02, 0.00)	-0.02 (-0.07, 0.02)	-0.01 (-0.03, 0.01)	-0.03 (-0.06, 0.01)
	TyG-WHtR	-0.00 (-0.01, 0.00)	-0.01 (-0.02, 0.00)	-0.00 (-0.01, 0.00)	-0.01 (-0.02, 0.00)
	TyG-WWI	-0.04 (-0.08, 0.00)	**-0.16 (-0.28**, -0.03)*	-0.03 (-0.09, 0.02)	-0.07 (-0.17, 0.03)
	TyG-ABSI	-0.48 (-1.00, 0.05)	-1.63 (-3.39, 0.13)	-0.29 (-1.04, 0.47)	-0.77 (-2.20, 0.65)

Notes: MoCA, Montreal Cognitive Assessment; DSST, Digit Symbol Substitution Test; AVLT-3, Auditory Verbal Learning Test-Immediate Recall Trial 3; AVLT-5, Auditory Verbal Learning Test-Delayed Recall; CI, confidence interval; TyG, triglyceride-glucose index; WHtR, waist-to-height ratio; BMI, body mass index; WC, waist circumference; WWI, weight-adjusted waist index; ABSI, a body shape index

Subgroup analyses were performed by gender, age (< 60 years/≥60 years) and BMI status (non-overweight: BMI < 24 kg/m²; overweight: BMI ≥ 24 kg/m²)

Adjusted for gender and age

* *p* < 0.05; ** *p* < 0.01

Sensitivity analyses were conducted by excluding participants with medication use, diabetes, or cardiovascular disease (Supplementary Table S9). The associations between TyG-related indices and cognitive outcomes remained largely consistent in direction and magnitude, indicating the robustness of the primary findings.

In addition, analyses based on multiple imputation for missing values yielded similar results, with no substantial changes in effect estimates (Supplementary Table S10).

Independent and joint effects of the TyG index and obesity-related indices were evaluated using three parallel models (Supplementary Table S11). In Model A, the TyG index was not significantly associated with cognitive outcomes across all tests. In Model B, several obesity-related indices, including WC, WHtR, WWI, and ABSI, showed inverse associations with MoCA scores. However, in Model C, when TyG and obesity indices were included simultaneously, these associations were attenuated and no longer statistically significant. Overall, neither TyG nor obesity-related indices demonstrated independent associations with cognitive outcomes after mutual adjustment.

Logistic regression analyses were performed to evaluate the clinical relevance of TyG-related indices using MCI (MoCA < 26) as the outcome (Supplementary Table S12). Neither the TyG index nor TyG-based obesity indices were significantly associated with the odds of MCI in either Model 1 or Model 2, with all odds ratios close to 1.

Model fit and predictive performance for different exposure indices are presented in Supplementary Table S13. Overall, TyG-based obesity indices showed slightly improved model fit compared with the TyG index alone, as reflected by marginally higher adjusted R² values and lower AIC and BIC across cognitive outcomes. Among these indices, TyG-WWI and TyG-WHtR generally demonstrated the best performance; however, the differences were small.

Variance inflation factor (VIF) analyses indicated no evidence of multicollinearity, with all VIF values below 5 across models (Supplementary Table S14).

In addition, no substantial ceiling effects were observed for cognitive outcome variables, with ceiling ratios below 15% for all tests (Supplementary Table S15).

## Discussion

This study demonstrated that elevated levels of the TyG index and its composite obesity indices including TyG-BMI, TyG-WC, TyG-WHtR, TyG-WWI, and TyG-ABSI were significantly associated with lower cognitive function. Furthermore, non-linear associations were observed between TyG-WWI, TyG-ABSI and global cognitive performance. In addition, stratified and sensitivity analyses showed generally consistent patterns, supporting the robustness of these findings. However, independent and joint analyses did not support clear independent effects of TyG and obesity-related indices after mutual adjustment, and no significant associations were observed when cognitive impairment was defined dichotomously. Despite the relatively small effect sizes, our findings still have important clinical and public health implications. Furthermore, given the high prevalence of insulin resistance and obesity, small individual-level effects may translate into a considerable population-level burden. Therefore, our findings support the potential value of incorporating TyG-related metabolic indices into routine health assessments for early risk stratification of cognitive aging. These findings suggest that the TyG index and its obesity-adjusted derivatives are associated with cognitive impairment, indicating their potential relevance in risk assessment.

Previous studies have reported inconsistent findings regarding the association between IR and cognitive function. For instance, Zhang et al. [25] reported that higher TyG index levels were associated with delayed cognitive decline and reduced risk of Alzheimer’s disease (AD), a finding that contradicts with our findings. However, recent evidence from the National Health and Nutrition Examination Survey (NHANES) 2011–2014 found no significant association between the homeostasis model assessment of IR and cognitive function in older adults [26]. Similarly, a prospective study in non-diabetic individuals found no significant link between IR levels and cognitive function, either cross-sectionally or longitudinally [27]. Notably, a growing body of evidence supports our results, showing that elevated TyG index is independently associated with cognitive impairment [8, 28–30]. These discrepancies may stem from differences in study populations, cognitive assessment tools, statistical methodologies, and the extent of covariate adjustment.

Although the underlying mechanisms linking the TyG index and related obesity indices to cognitive function are not fully elucidated, several plausible biological pathways have been proposed. First, IR impairs cerebral glucose metabolism [31], leading to neuronal energy deficits and promoting the accumulation of neurotoxic proteins such as amyloid - β, thereby contributing to cognitive decline [32]. Triglycerides can cross the blood-brain barrier and may induce central IR, further impairing synaptic plasticity and cognitive function [33]. Second, IR is associated with endothelial dysfunction, atherosclerosis, and increased arterial stiffness [34], which compromise cerebrovascular reactivity and impair clearance of metabolic waste via the glymphatic system [35]. These vascular insults may initiate neuroinflammatory cascades that exacerbate neurodegeneration [36].

Notably, when TyG and obesity-related indices were included simultaneously, the observed associations were attenuated, suggesting that these effects may reflect shared or overlapping metabolic pathways rather than independent contributions.

Furthermore, the non-linear association observed for TyG-WWI suggests that the relationship between metabolic burden and cognitive function may vary across exposure levels. The presence of a threshold effect indicates a steeper decline at lower levels followed by a plateau, highlighting the importance of considering non-linear relationships when evaluating metabolic risk factors and cognitive outcomes.

Our study revealed a gender-specific association, with stronger inverse relationships between metabolic indices and cognitive function observed in women. This finding is consistent with prior studies suggesting greater vulnerability of women to IR-related cognitive decline [8, 37]. Laws et al. [38] reported higher prevalence of insulin resistance in women compared to men, although some studies have found no significant association in women [39] or even stronger associations in men [40]. Given the mean age of our female participants (49.4 years), one plausible explanation involves the perimenopausal transition—a period characterized by declining estrogen, a hormone with neuroprotective properties [41–43]. This physiological change may render women more susceptible to the cognitive impacts of metabolic dysregulation. Socioeconomic and lifestyle factors may further compound this vulnerability [44]. In age-stratified analyses, the associations were significant only among participants under 60 years, possibly reflecting the higher burden of competing risks—such as age-related neurodegeneration, multimorbidity, and cumulative vascular damage—in older adults [2]. Additionally, the increasing prevalence of IR in younger populations due to modifiable factors like sedentary behavior and excess energy intake may contribute to the observed age pattern [45].Notably, these subgroup findings should be interpreted with caution, as formal interaction analyses did not detect consistent or statistically significant effect modification across strata. Additionally, our study lacked data on menopausal status and hormone replacement therapy. Therefore, the hypothesized role of hormonal changes in driving the observed gender difference warrants direct, targeted investigation in future studies.

Interestingly, no significant associations were observed when cognitive impairment was defined dichotomously. This may be partly explained by the loss of information and reduced statistical power associated with dichotomization, as well as the relatively low prevalence of impairment in this population. These findings suggest that TyG-related indices may be more sensitive to subtle variations in cognitive performance rather than clinically defined impairment.

In addition, although TyG-based obesity indices showed slightly improved model fit compared with the TyG index alone, the magnitude of improvement was modest, suggesting limited incremental clinical utility.

The strengths of this study include the use of multiple validated neuropsychological tests and comprehensive assessment of both the TyG index and its obesity-adjusted composite indices. However, several limitations warrant acknowledgment. First, the cross-sectional design precludes causal inference and temporal confirmation between TyG-related indices and cognitive function. Reverse causality remains plausible, as cognitive impairment may affect the metabolic and behavioral factors linked to these indices. Future studies using Mendelian randomization or prospective longitudinal designs are required to verify the causal association. Second, our participants were recruited from a single health check-up center, which may introduce healthy user bias. Individuals attending voluntary health check-ups tend to have higher socioeconomic status, better health awareness, and healthier lifestyle behaviors compared to the general population. This bias may lead to an underestimation of the strength of the associations between TyG-related indices and cognitive function. Third, our single-center design with participants recruited primarily from Dongyang City limits the generalizability of our findings to other populations. Future validation in multi-center cohorts or public databases (e.g., NHANES, KNHANES, CHARLS) is warranted. Finally, despite adjustment for multiple confounders, residual confounding due to unmeasured factors–including dietary patterns, sleep quality, depressive symptoms, serum phosphate levels, environmental factors, and detailed medication history–cannot be entirely excluded.

In conclusion, the study demonstrates that elevated TyG index and related obesity indices are associated with worse cognitive function in healthy middle-aged Chinese adults. These findings underscore the potential association between metabolic health and cognitive aging, and support the integration of metabolic biomarkers into early risk assessment for cognitive decline. Future longitudinal studies are needed to confirm causality and evaluate the utility of early metabolic interventions in preserving cognitive function.

## Electronic Supplementary Material

Below is the link to the electronic supplementary material.

Supplementary Material 1: Figure S1. Restricted cubic spline (RCS) analyses of the associations between TyG and related obesity indices with DSSTa. Panels A-F: RCS curves for TyG, TyG-BMI, TyG-WC, TyG-WHtR, TyG-WWI, and TyG-ABSI and DSST. a Adjusted for gender, age, education level, alcohol consumption, smoking status, body mass index (BMI), total cholesterol, physical activity, and history of hypertension. DSST, Digit Symbol Substitution Test; TyG, triglyceride-glucose index; TyG-BMI, triglyceride glucose-body mass index; TyG-WC, triglyceride glucose-waist circumference; TyG-WHtR, triglyceride glucose-waist-to-height ratio; TyG-WWI, Triglyceride-Glucose Waist-to-Weight Index; TyG-ABSI, Triglyceride-Glucose-A Body Shape Index.

Supplementary Material 2: Figure S2. Restricted cubic spline (RCS) analyses of the associations between TyG and related obesity indices with AVLT-3a. Panels A-F: RCS curves for TyG, TyG-BMI, TyG-WC, TyG-WHtR, TyG-WWI, and TyG-ABSI and AVLT-3. a Adjusted for gender, age, education level, alcohol consumption, smoking status, body mass index (BMI), total cholesterol, physical activity, and history of hypertension. AVLT-3, Auditory Verbal Learning Test-Immediate Recall Trial 3; TyG, triglyceride-glucose index; TyG-BMI, triglyceride glucose-body mass index; TyG-WC, triglyceride glucose-waist circumference; TyG-WHtR, triglyceride glucose-waist-to-height ratio; TyG-WWI, Triglyceride-Glucose Waist-to-Weight Index; TyG-ABSI, Triglyceride-Glucose-A Body Shape Index.

Supplementary Material 3: Figure S3. Restricted cubic spline (RCS) analyses of the associations between TyG and related obesity indices with AVLT-5a. Panels A-F: RCS curves for TyG, TyG-BMI, TyG-WC, TyG-WHtR, TyG-WWI, and TyG-ABSI and AVLT-5. a Adjusted for gender, age, education level, alcohol consumption, smoking status, body mass index (BMI), total cholesterol, physical activity, and history of hypertension. AVLT-5, Auditory Verbal Learning Test-Delayed Recall; TyG, triglyceride-glucose index; TyG-BMI, triglyceride glucose-body mass index; TyG-WC, triglyceride glucose-waist circumference; TyG-WHtR, triglyceride glucose-waist-to-height ratio; TyG-WWI, Triglyceride-Glucose Waist-to-Weight Index; TyG-ABSI, Triglyceride-Glucose-A Body Shape Index.

Supplementary Material 4

Supplementary Material 5

Supplementary Material 6

Supplementary Material 7

Supplementary Material 8

Supplementary Material 9

Supplementary Material 10

Supplementary Material 11

Supplementary Material 12

Supplementary Material 13

Supplementary Material 14

Supplementary Material 15

Supplementary Material 16

Supplementary Material 17

Supplementary Material 18

## Data Availability

data described in the manuscript will be available upon reasonable re-quest pending approval by the corresponding authors.

## Declarations

### Declarations of generative AI and AI-assisted technologies in the writing process

No generative AI or AI-assisted technologies were used in the preparation of this manuscript.
