# Supplementary figures and images for: Association of TyG index and obesity indicators with cognitive function: a cross - sectional study from Chinese health check-up centers
Source: BMC Endocr Disord. 2026 Apr 17;26:169. doi: 10.1186/s12902-026-02280-4 (PMC13224721; doi:10.1186/s12902-026-02280-4)

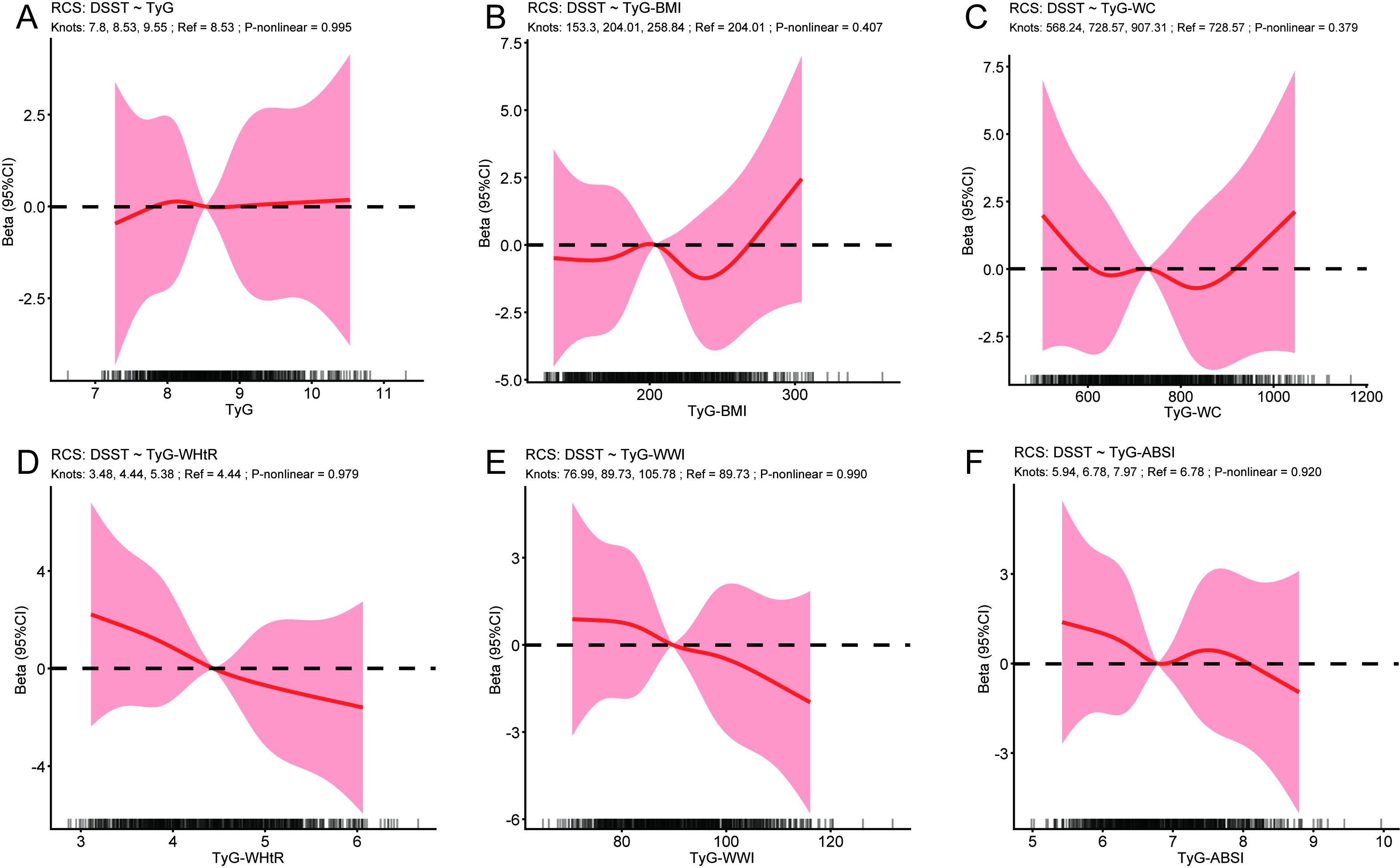

Supplement: Supplementary file 1 — Supplementary Material 1: Figure S1. Restricted cubic spline (RCS) analyses of the associations between TyG and related obesity indices with DSSTa. Panels A-F: RCS curves for TyG, TyG-BMI, TyG-WC, TyG-WHtR, TyG-WWI, and TyG-ABSI and DSST. a Adjusted for gender, age, education level, alcohol consumption, smoking status, body mass index (BMI), total cholesterol, physical activity, and history of hypertension. DSST, Digit Symbol Substitution Test; TyG, triglyceride-glucose index; TyG-BMI, triglyceride glucose-body mass index; TyG-WC, triglyceride glucose-waist circumference; TyG-WHtR, triglyceride glucose-waist-to-height ratio; TyG-WWI, Triglyceride-Glucose Waist-to-Weight Index; TyG-ABSI, Triglyceride-Glucose-A Body Shape Index. [file 12902_2026_2280_MOESM1_ESM.tif]

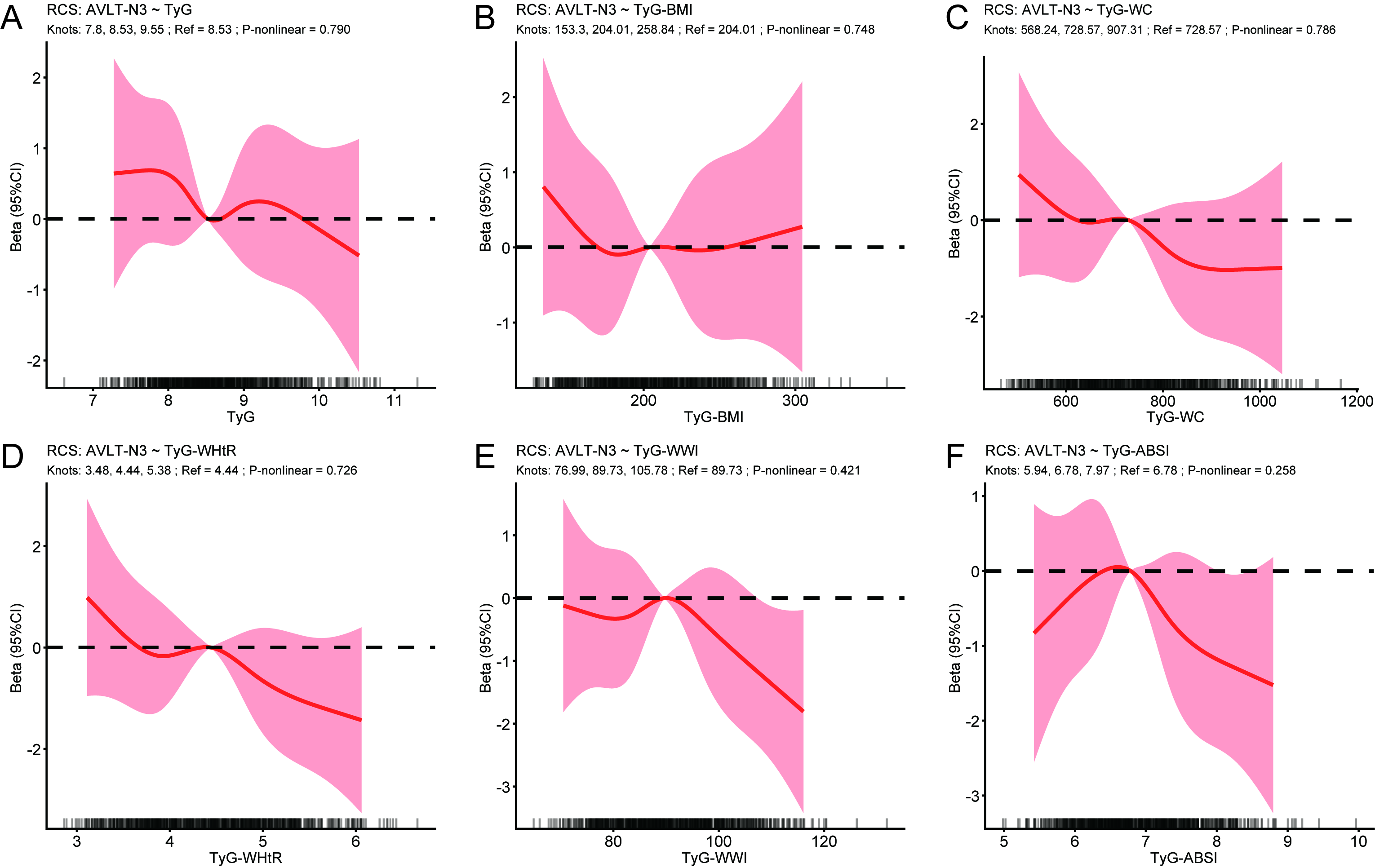

Supplement: Supplementary file 2 — Supplementary Material 2: Figure S2. Restricted cubic spline (RCS) analyses of the associations between TyG and related obesity indices with AVLT-3a. Panels A-F: RCS curves for TyG, TyG-BMI, TyG-WC, TyG-WHtR, TyG-WWI, and TyG-ABSI and AVLT-3. a Adjusted for gender, age, education level, alcohol consumption, smoking status, body mass index (BMI), total cholesterol, physical activity, and history of hypertension. AVLT-3, Auditory Verbal Learning Test-Immediate Recall Trial 3; TyG, triglyceride-glucose index; TyG-BMI, triglyceride glucose-body mass index; TyG-WC, triglyceride glucose-waist circumference; TyG-WHtR, triglyceride glucose-waist-to-height ratio; TyG-WWI, Triglyceride-Glucose Waist-to-Weight Index; TyG-ABSI, Triglyceride-Glucose-A Body Shape Index. [file 12902_2026_2280_MOESM2_ESM.tif]

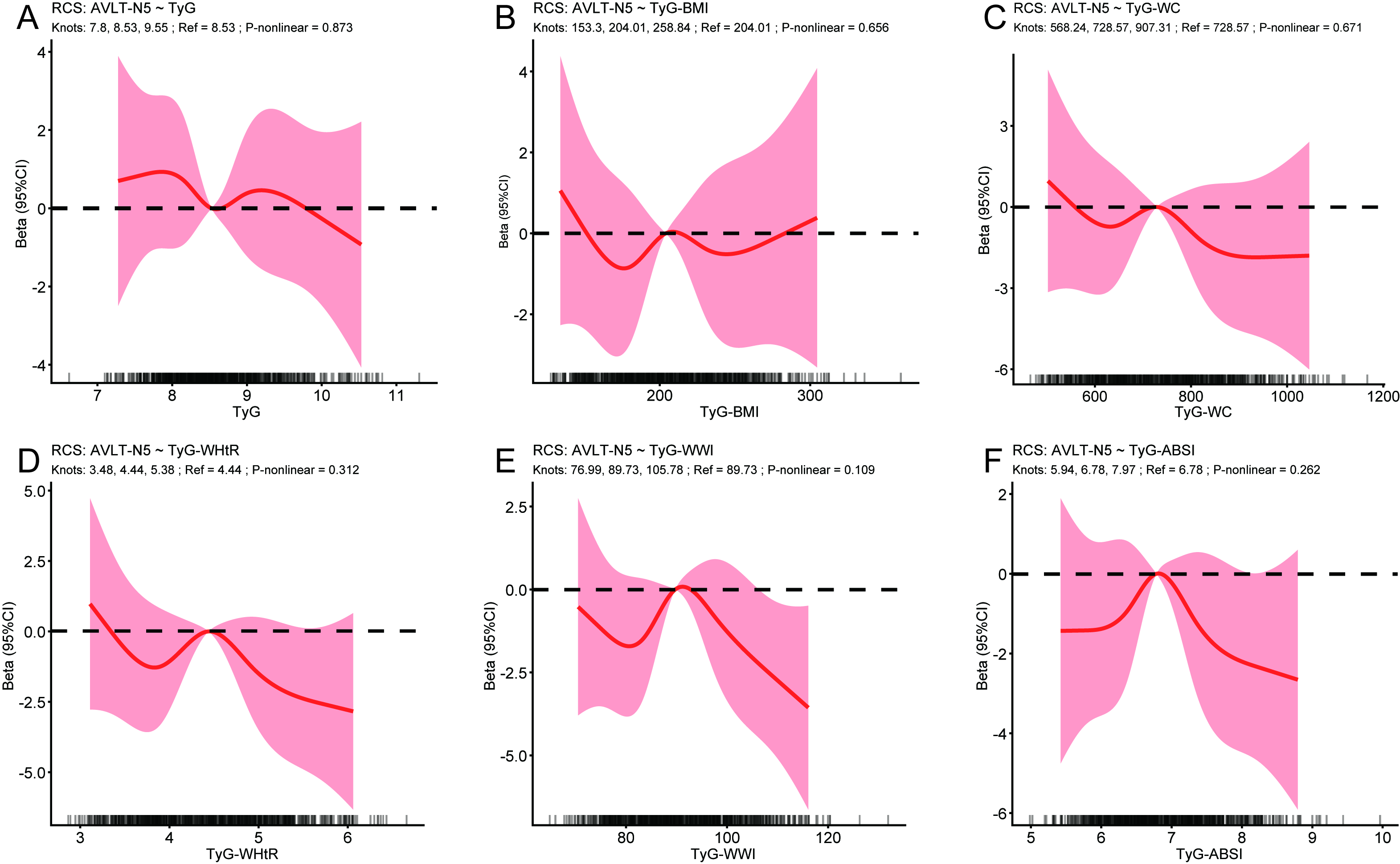

Supplement: Supplementary file 3 — Supplementary Material 3: Figure S3. Restricted cubic spline (RCS) analyses of the associations between TyG and related obesity indices with AVLT-5a. Panels A-F: RCS curves for TyG, TyG-BMI, TyG-WC, TyG-WHtR, TyG-WWI, and TyG-ABSI and AVLT-5. a Adjusted for gender, age, education level, alcohol consumption, smoking status, body mass index (BMI), total cholesterol, physical activity, and history of hypertension. AVLT-5, Auditory Verbal Learning Test-Delayed Recall; TyG, triglyceride-glucose index; TyG-BMI, triglyceride glucose-body mass index; TyG-WC, triglyceride glucose-waist circumference; TyG-WHtR, triglyceride glucose-waist-to-height ratio; TyG-WWI, Triglyceride-Glucose Waist-to-Weight Index; TyG-ABSI, Triglyceride-Glucose-A Body Shape Index. [file 12902_2026_2280_MOESM3_ESM.tif]
