# Supplementary material for: Association of TyG index and obesity indicators with cognitive function: a cross - sectional study from Chinese health check-up centers
Source: BMC Endocr Disord. 2026 Apr 17;26:169. doi: 10.1186/s12902-026-02280-4 (PMC13224721; doi:10.1186/s12902-026-02280-4)
Supplement: Supplementary file 4 — Supplementary Material 4 [file 12902_2026_2280_MOESM4_ESM.docx]

### Table S1. Baseline characteristics of the study population. (AVLT)

| Characteristics | Overall  (N=719) | Triglyceride-glucose index | | | | *P value* |
| --- | --- | --- | --- | --- | --- | --- |
|  |  | Quartile 1 (N=180) | Quartile 2 (N=176) | Quartile 3 (N=181) | Quartile 4 (N=182) |  |
| Age, years (mean (SD)) | 49.4 (13.4) | 45.5 (14.2) | 50.5 (13.8) | 50.6 (13.3) | 51.2 (11.2) | <0.001 |
| gender, female, n (%) | 322 (44.8) | 113 (62.8) | 89 (50.6) | 74 (40.9) | 46 (25.3) | <0.001 |
| Education level, n (%) |  |  |  |  |  | 0.167 |
| Less than primary school | 61 (8.5) | 12 (6.7) | 15 (8.5) | 18 (9.9) | 16 (8.8) |  |
| High school or equivalent | 302 (42.0) | 66 (36.7) | 71 (40.3) | 75 (41.4) | 90 (49.5) |  |
| College or above | 356 (49.5) | 102 (56.7) | 90 (51.1) | 88 (48.6) | 76 (41.8) |  |
| Alcohol = yes, n (%) | 299 (41.6) | 49 (27.2) | 66 (37.5) | 85 (47.0) | 99 (54.4) | <0.001 |
| Smoking = yes, n (%) | 196 (27.3) | 29 (16.1) | 39 (22.2) | 56 (30.9) | 72 (39.6) | <0.001 |
| BMI (mean (SD)) | 23.9 (3.5) | 21.5 (2.9) | 23.3 (3.1) | 25.0 (3.3) | 25.7 (3.0) | <0.001 |
| Activity = low, n (%) | 81 (11.3) | 18 (10.0) | 19 (10.8) | 19 (10.5) | 25 (13.7) | 0.672 |
| TC (mean (SD)) | 4.9 (1.0) | 4.6 (0.9) | 4.8 (0.9) | 4.9 (0.9) | 5.2 (1.0) | <0.001 |
| Hypertension = Yes, n (%) | 143 (19.9) | 11 (6.1) | 29 (16.5) | 40 (22.1) | 63 (34.6) | <0.001 |
| AVLT_N3, mean (SD) | 17.9 (5.6) | 19.4 (5.2) | 18.0 (5.9) | 17.5 (5.4) | 16.9 (5.6) | <0.001 |
| AVLT_N5, mean (SD) | 30.5 (10.6) | 33.0 (9.8) | 30.5 (11.1) | 29.6 (10.0) | 28.8 (10.9) | 0.001 |
| TyG-BMI (mean (SD)) | 207.0 (40.2) | 168.2 (24.1) | 194.1 (25.3) | 218.7 (29.5) | 246.3 (31.8) | <0.001 |
| TyG-WC (mean (SD)) | 736.3 (132.8) | 604.4 (72.0) | 692.9 (77.1) | 770.2 (96.3) | 875.2 (100.6) | <0.001 |
| TyG-WHtR (mean (SD)) | 4.5 (0.8) | 3.7 (0.4) | 4.2 (0.5) | 4.7 (0.5) | 5.2 (0.5) | <0.001 |
| TyG-WWI (mean (SD)) | 90.9 (11.0) | 79.3 (5.5) | 87.4 (6.3) | 93.3 (6.9) | 103.3 (7.8) | <0.001 |
| TyG-ABSI (mean (SD)) | 6.9 (0.8) | 6.1 (0.4) | 6.6 (0.4) | 7.0 (0.5) | 7.8 (0.6) | <0.001 |

Note: SD, standard deviation; TC, total cholesterol; MoCA, Montreal Cognitive Assessment; AVLT-3, Auditory Verbal Learning Test-Immediate Recall Trial 3; AVLT-5, Auditory Verbal Learning Test-Delayed Recall; TyG, triglyceride-glucose index; WHtR, waist-to-height ratio; BMI, body mass index; WC, waist circumference; WWI, weight-adjusted waist index; ABSI, a body shape index.

Continuous variables were presented as mean (SD); categorical variables were presented as numbers (percentages). Group differences were compared using Analysis of Variance (ANOVA) and the chi-square test, respectively. Percentages may not total 100 due to rounding.
