# Supplementary material for: Association of TyG index and obesity indicators with cognitive function: a cross - sectional study from Chinese health check-up centers
Source: BMC Endocr Disord. 2026 Apr 17;26:169. doi: 10.1186/s12902-026-02280-4 (PMC13224721; doi:10.1186/s12902-026-02280-4)
Supplement: Supplementary file 5 — Supplementary Material 5 [file 12902_2026_2280_MOESM5_ESM.docx]

### Table S2. Baseline characteristics of the study population (DSST).

| Characteristics | Overall  (N=713) | Triglyceride-glucose index | | | | *P* | |
| --- | --- | --- | --- | --- | --- | --- | --- |
|  |  | Quartile 1 (N=18o) | Quartile 2 (N=175) | Quartile 3 (N=181) | Quartile 4 (N=177) |  |  |
| Age, years (mean (SD)) | 49.4 (13.3) | 45.5 (14.2) | 50.4 (13.8) | 50.6 (13.3) | 51.0 (11.0) | <0.001 |  |
| gender, female, n (%) | 320 (44.9) | 113 (62.8) | 88 (50.3) | 74 (40.9) | 45 (25.4) | <0.001 |  |
| Education level, n (%) |  |  |  |  |  | 0.205 |  |
| Less than primary school | 59 (8.3) | 12 (6.7) | 14 (8.0) | 18 (9.9) | 15 (8.5) |  |  |
| High school or equivalent | 299 (41.9) | 66 (36.7) | 71 (40.6) | 75 (41.4) | 87 (49.2) |  |  |
| College or above | 355 (49.8) | 102 (56.7) | 90 (51.4) | 88 (48.6) | 75 (42.4) |  |  |
| Alcohol = yes, n (%) | 297 (41.7) | 49 (27.2) | 66 (37.7) | 85 (47.0) | 97 (54.8) | <0.001 |  |
| Smoking = yes, n (%) | 193 (27.1) | 29 (16.1) | 39 (22.3) | 56 (30.9) | 69 (39.0) | <0.001 |  |
| BMI (mean (SD)) | 23.9 (3.5) | 21.5 (2.9) | 23.3 (3.1) | 25.0 (3.3) | 25.8 (3.1) | <0.001 |  |
| Activity = low, n (%) | 80 (11.2) | 18 (10.0) | 19 (10.9) | 19 (10.5) | 24 (13.6) | 0.715 |  |
| TC (mean (SD)) | 4.9 (1.0) | 4.6 (0.9) | 4.8 (0.9) | 4.9 (0.9) | 5.2 (1.0) | <0.001 |  |
| Hypertension = Yes, n (%) | 140 (19.6) | 11 (6.1) | 29 (16.6) | 40 (22.1) | 60 (33.9) | <0.001 |  |
| DSST, mean (SD) | 51.1 (17.9) | 56.1 (20.0) | 50.5 (18.0) | 50.1 (16.9) | 47.6 (15.5) | <0.001 |  |
| TyG-BMI (mean (SD)) | 206.8 (40.3) | 168.2 (24.1) | 193.9 (25.2) | 218.7 (29.5) | 246.6 (32.1) | <0.001 |  |
| TyG-WC (mean (SD)) | 735.3 (132.8) | 604.4 (72.0) | 692.1 (76.6) | 770.2 (96.3) | 875.7 (101.4) | <0.001 |  |
| TyG-WHtR (mean (SD)) | 4.4 (0.8) | 3.7 (0.4) | 4.2 (0.5) | 4.7 (0.5) | 5.2 (0.5) | <0.001 |  |
| TyG-WWI (mean (SD)) | 90.8 (11.0) | 79.3 (5.5) | 87.4 (6.2) | 93.3 (6.9) | 103.4 (7.9) | <0.001 |  |
| TyG-ABSI (mean (SD)) | 6.9 (0.8) | 6.1 (0.4) | 6.6 (0.4) | 7.0 (0.5) | 7.8 (0.6) | <0.001 |  |

Note: SD, standard deviation; TC, total cholesterol; DSST, Digit Symbol Substitution Test; TyG, triglyceride-glucose index; WHtR, waist-to-height ratio; BMI, body mass index; WC, waist circumference; WWI, weight-adjusted waist index; ABSI, a body shape index.

Continuous variables were presented as mean (SD); categorical variables were presented as numbers (percentages). Group differences were compared using Analysis of Variance (ANOVA) and the chi-square test, respectively. Percentages may not total 100 due to rounding.
