# Supplementary material for: Association of TyG index and obesity indicators with cognitive function: a cross - sectional study from Chinese health check-up centers
Source: BMC Endocr Disord. 2026 Apr 17;26:169. doi: 10.1186/s12902-026-02280-4 (PMC13224721; doi:10.1186/s12902-026-02280-4)
Supplement: Supplementary file 6 — Supplementary Material 6 [file 12902_2026_2280_MOESM6_ESM.docx]

Table S3. Association of TyG and related obesity indices with cognitive function (Standardized beta).

| **Outcome** | **Exposure** | **Model 1** | | **Model 2** | |
| --- | --- | --- | --- | --- | --- |
|  |  | **Beta (95%CI)** | ***P* value** | **Beta (95%CI)** | ***P* value** |
| **MoCA** | TyG | -0.08 (-0.15, -0.01) | 0.102 | -0.03 (-0.10, 0.03) | 0.406 |
|  | TyG-BMI | -0.09 (-0.16, -0.02) | 0.085 | 0.01 (-0.10, 0.11) | 0.923 |
|  | TyG-WC | **-0.12 (-0.19, -0.04)** | **0.035** | -0.12 (-0.22, -0.01) | 0.118 |
|  | TyG-WHtR | **-0.12 (-0.19, -0.05)** | **0.022** | -0.11 (-0.21, -0.01) | 0.119 |
|  | TyG-WWI | **-0.12 (-0.19, -0.05)** | **0.022** | -0.08 (-0.15, -0.01) | 0.118 |
|  | TyG-ABSI | **-0.11 (-0.18, -0.04)** | **0.032** | -0.08 (-0.14, -0.01) | 0.118 |
| **DSST** | TyG | -0.03 (-0.08, 0.02) | 0.328 | 0.00 (-0.05, 0.06) | 0.929 |
|  | TyG-BMI | -0.04 (-0.10, 0.01) | 0.23 | 0.02 (-0.06, 0.10) | 0.768 |
|  | TyG-WC | -0.05 (-0.11, 0.01) | 0.212 | -0.01 (-0.09, 0.07) | 0.859 |
|  | TyG-WHtR | -0.07 (-0.12, -0.02) | 0.08 | -0.05 (-0.13, 0.02) | 0.293 |
|  | TyG-WWI | -0.07 (-0.13, -0.02) | 0.068 | -0.04 (-0.09, 0.02) | 0.288 |
|  | TyG-ABSI | -0.06 (-0.11, -0.00) | 0.142 | -0.02 (-0.08, 0.03) | 0.454 |
| **AVLT-3** | TyG | -0.06 (-0.12, 0.01) | 0.212 | -0.04 (-0.11, 0.03) | 0.356 |
|  | TyG-BMI | -0.06 (-0.12, 0.01) | 0.224 | -0.01 (-0.12, 0.10) | 0.886 |
|  | TyG-WC | -0.07 (-0.15, 0.00) | 0.156 | -0.09 (-0.20, 0.02) | 0.23 |
|  | TyG-WHtR | -0.08 (-0.15, -0.01) | 0.102 | -0.10 (-0.20, 0.01) | 0.181 |
|  | TyG-WWI | -0.09 (-0.15, -0.02) | 0.08 | -0.07 (-0.15, 0.00) | 0.169 |
|  | TyG-ABSI | -0.08 (-0.15, -0.01) | 0.118 | -0.07 (-0.14, 0.01) | 0.181 |
| **AVLT-5** | TyG | -0.04 (-0.11, 0.02) | 0.308 | -0.03 (-0.10, 0.04) | 0.495 |
|  | TyG-BMI | -0.05 (-0.12, 0.02) | 0.252 | 0.00 (-0.11, 0.11) | 0.987 |
|  | TyG-WC | -0.07 (-0.14, 0.01) | 0.181 | -0.08 (-0.20, 0.03) | 0.248 |
|  | TyG-WHtR | -0.07 (-0.14, -0.01) | 0.133 | -0.09 (-0.19, 0.02) | 0.224 |
|  | TyG-WWI | -0.07 (-0.14, -0.01) | 0.12 | -0.06 (-0.14, 0.01) | 0.224 |
|  | TyG-ABSI | -0.07 (-0.14, 0.00) | 0.173 | -0.06 (-0.13, 0.02) | 0.231 |

Notes: MoCA, Montreal Cognitive Assessment; DSST, Digit Symbol Substitution Test; AVLT-3, Auditory Verbal Learning Test-Immediate Recall Trial 3; AVLT-5, Auditory Verbal Learning Test-Delayed Recall; CI, confidence interval; TyG, triglyceride-glucose index; WHtR, waist-to-height ratio; BMI, body mass index; WC, waist circumference; WWI, weight-adjusted waist index; ABSI, a body shape index.

Model 1 Adjusted for gender and age

Model 2 Adjusted for gender, age, education level, alcohol consumption, smoking status, BMI, WC, total cholesterol, physical activity, and history of hypertension. The 95% CIs are unadjusted; p-values are FDR-adjusted using the Benjamini–Hochberg procedure. To avoid over-adjustment bias, the corresponding anthropometric component was excluded from covariates in models for each composite index
