# Supplementary material for: Association of TyG index and obesity indicators with cognitive function: a cross - sectional study from Chinese health check-up centers
Source: BMC Endocr Disord. 2026 Apr 17;26:169. doi: 10.1186/s12902-026-02280-4 (PMC13224721; doi:10.1186/s12902-026-02280-4)
Supplement: Supplementary file 7 — Supplementary Material 7 [file 12902_2026_2280_MOESM7_ESM.docx]

Table S4.Association of TyG and its composite indices quartiles with cognitive function (Model2).

|  | **MOCA**  **Beta (95%CI)** | **DSST**  **Beta (95%CI)** | **AVLT-N3**  **Beta (95%CI)** | **AVLT-N5**  **Beta (95%CI)** |
| --- | --- | --- | --- | --- |
| TyG |  |  |  |  |
| Quartile 1 | **Ref.** |  |  |  |
| Quartile 2 | -0.31 (-0.91, 0.30) | -0.27 (-1.21, 0.68) | -0.26 (-2.10, 1.57) | -0.65 (-2.89, 1.59) |
| Quartile 3 | 0.01 (-0.64, 0.66) | -0.53 (-1.54, 0.49) | -0.70 (-2.65, 1.25) | 0.41 (-1.98, 2.80) |
| Quartile 4 | -0.43 (-1.12, 0.26) | -0.69 (-1.77, 0.40) | -0.9 (-2.99, 1.18) | -0.44 (-3.00, 2.12) |
| TyG-BMI |  |  |  |  |
| Quartile 1 | **Ref.** |  |  |  |
| Quartile 2 | -0.38 (-1.09, 0.32) | -0.67 (-1.77, 0.43) | -0.15 (-2.28, 1.97) | -0.01 (-2.6, 2.58) |
| Quartile 3 | -0.53 (-1.42, 0.35) | -0.83 (-2.21, 0.55) | -0.83 (-3.5, 1.83) | -0.38 (-3.66, 2.89) |
| Quartile 4 | -0.52 (-1.71, 0.67) | -0.56 (-2.42, 1.29) | -0.10 (-3.68, 3.49) | -0.60 (-5.02, 3.82) |
| TyG-WC |  |  |  |  |
| Quartile 1 | **Ref.** |  |  |  |
| Quartile 2 | -0.19 (-0.84, 0.46) | 0.22 (-0.8, 1.25) | 0.46 (-1.52, 2.43) | -0.14 (-2.55, 2.27) |
| Quartile 3 | -0.74 (-1.54, 0.07) | -0.21 (-1.48, 1.06) | 0.17 (-2.27, 2.61) | -1.06 (-4.04, 1.92) |
| Quartile 4 | **-1.03 (-2.02, -0.03)*** | -0.59 (-2.15, 0.97) | -0.97 (-3.96, 2.02) | -0.07 (-3.75, 3.61) |
| TyG-WHtR |  |  |  |  |
| Quartile 1 | **Ref.** |  |  |  |
| Quartile 2 | -0.25 (-0.9, 0.39) | -0.53 (-1.55, 0.49) | -0.57 (-2.52, 1.39) | -1.41 (-3.80, 0.98) |
| Quartile 3 | -0.04 (-0.80, 0.72) | -0.45 (-1.64, 0.75) | -0.04 (-2.33, 2.25) | -2.11 (-4.93, 0.71) |
| Quartile 4 | **-0.97 (-1.89, -0.06)*** | **-1.53 (-2.97, -0.10)*** | -2.26 (-5.02, 0.50) | -2.50 (-5.89, 0.89) |
| TyG-WWI |  |  |  |  |
| Quartile 1 | **Ref.** |  |  |  |
| Quartile 2 | 0.32 (-0.28, 0.92) | -0.18 (-1.12, 0.76) | 0.21 (-1.61, 2.03) | -0.21 (-2.43, 2.02) |
| Quartile 3 | 0.39 (-0.28, 1.06) | 0.18 (-0.88, 1.24) | 1.09 (-0.94, 3.12) | -0.81 (-3.31, 1.69) |
| Quartile 4 | -0.64 (-1.36, 0.08) | -0.90 (-2.03, 0.23) | -1.15 (-3.32, 1.01) | -1.43 (-4.11, 1.25) |
| TyG-ABSI |  |  |  |  |
| Quartile 1 | **Ref.** |  |  |  |
| Quartile 2 | 0.30 (-0.30, 0.91) | 0.47 (-0.48, 1.41) | 0.86 (-0.98, 2.69) | -0.15 (-2.39, 2.09) |
| Quartile 3 | 0.05 (-0.59, 0.70) | 0.00 (-1.01, 1.01) | 0.68 (-1.27, 2.64) | -0.79 (-3.20, 1.62) |
| Quartile 4 | -0.58 (-1.29, 0.12) | -0.81 (-1.91, 0.29) | -1.35 (-3.47, 0.76) | -0.86 (-3.47, 1.75) |

Notes: MoCA, Montreal Cognitive Assessment; DSST, Digit Symbol Substitution Test; AVLT-3, Auditory Verbal Learning Test-Immediate Recall Trial 3; AVLT-5, Auditory Verbal Learning Test-Delayed Recall; CI, confidence interval; TyG, triglyceride-glucose index; WHtR, waist-to-height ratio; BMI, body mass index; WC, waist circumference; WWI, weight-adjusted waist index; ABSI, a body shape index.

Adjusted for gender, age, education level, alcohol consumption, smoking status, BMI, WC, total cholesterol, physical activity, and history of hypertension. To avoid over-adjustment bias, the corresponding anthropometric component was excluded from covariates in models for each composite index

* p < 0.05; ** p < 0.01; *** p < 0.001.
