# Supplementary material for: Association of TyG index and obesity indicators with cognitive function: a cross - sectional study from Chinese health check-up centers
Source: BMC Endocr Disord. 2026 Apr 17;26:169. doi: 10.1186/s12902-026-02280-4 (PMC13224721; doi:10.1186/s12902-026-02280-4)
Supplement: Supplementary file 8 — Supplementary Material 8 [file 12902_2026_2280_MOESM8_ESM.docx]

Table S5. Subgroup analyses of the Association between TyG with cognitive function (Model2)

|  |  | **MoCA**  **Beta (95%CI)** | **DSST**  **Beta (95%CI)** | **AVLT-3**  **Beta (95%CI)** | **AVLT-5**  **Beta (95%CI)** |
| --- | --- | --- | --- | --- | --- |
| **Gender** |  |  |  |  |  |
| Male | TyG | -0.20 (-0.61, 0.22) | 0.12 (-1.46, 1.71) | -0.44 (-1.12, 0.25) | -0.76 (-2.08, 0.55) |
|  | TyG-BMI | -0.00 (-0.02, 0.01) | 0.02 (-0.03, 0.06) | -0.01 (-0.03, 0.01) | -0.02 (-0.06, 0.02) |
|  | TyG-WC | -0.00 (-0.01, 0.00) | 0.00 (-0.01, 0.01) | -0.00 (-0.01, 0.00) | -0.01 (-0.02, 0.01) |
|  | TyG-WHtR | -0.31 (-0.89, 0.28) | -1.06 (-3.28, 1.16) | -0.50 (-1.46, 0.47) | -0.89 (-2.73, 0.95) |
|  | TyG-WWI | -0.02 (-0.05, 0.01) | -0.05 (-0.16, 0.06) | -0.03 (-0.07, 0.02) | -0.04 (-0.14, 0.05) |
|  | TyG-ABSI | -0.23 (-0.63, 0.16) | -0.38 (-1.87, 1.11) | -0.28 (-0.93, 0.37) | -0.57 (-1.81, 0.67) |
| Female | TyG | -0.07 (-0.71, 0.57) | 0.59 (-1.70, 2.89) | -0.05 (-1.01, 0.91) | 0.41 (-1.44, 2.26) |
|  | TyG-BMI | 0.01 (-0.01, 0.02) | 0.00 (-0.06, 0.06) | 0.02 (-0.01, 0.04) | 0.03 (-0.01, 0.08) |
|  | TyG-WC | -0.01 (-0.01, 0.00) | -0.00 (-0.02, 0.02) | -0.01 (-0.02, 0.00) | -0.01 (-0.03, 0.01) |
|  | TyG-WHtR | -0.86 (-1.73, 0.01) | -1.16 (-4.30, 1.99) | -1.03 (-2.36, 0.30) | -1.42 (-3.97, 1.14) |
|  | TyG-WWI | -0.04 (-0.08, 0.00) | -0.05 (-0.20, 0.10) | -0.05 (-0.11, 0.01) | -0.06 (-0.19, 0.06) |
|  | TyG-ABSI | -0.50 (-1.07, 0.07) | -0.43 (-2.47, 1.62) | -0.72 (-1.59, 0.14) | -0.81 (-2.48, 0.86) |
| **Age** |  |  |  |  |  |
| <60 Years | TyG | -0.16 (-0.51, 0.18) | -0.13 (-1.56, 1.31) | -0.22 (-0.85, 0.41) | -0.17 (-1.36, 1.02) |
|  | TyG-BMI | 0.00 (-0.01, 0.01) | 0.01 (-0.04, 0.05) | 0.00 (-0.02, 0.02) | 0.01 (-0.03, 0.04) |
|  | TyG-WC | -0.00 (-0.01, 0.00) | -0.00 (-0.01, 0.01) | -0.00 (-0.01, 0.00) | -0.00 (-0.01, 0.01) |
|  | TyG-WHtR | -0.36 (-0.84, 0.12) | -0.93 (-2.92, 1.05) | -0.52 (-1.39, 0.35) | -0.70 (-2.35, 0.94) |
|  | TyG-WWI | -0.02 (-0.04, 0.01) | -0.05 (-0.15, 0.05) | -0.03 (-0.07, 0.02) | -0.04 (-0.12, 0.05) |
|  | TyG-ABSI | -0.28 (-0.60, 0.03) | -0.53 (-1.85, 0.78) | -0.34 (-0.92, 0.24) | -0.43 (-1.53, 0.66) |
| ≥60 Years | TyG | -0.39 (-1.64, 0.87) | 2.44 (-1.08, 5.97) | -1.10 (-2.43, 0.23) | -2.10 (-4.75, 0.55) |
|  | TyG-BMI | -0.00 (-0.03, 0.03) | 0.04 (-0.05, 0.12) | -0.02 (-0.05, 0.02) | -0.03 (-0.10, 0.03) |
|  | TyG-WC | -0.01 (-0.02, 0.00) | 0.01 (-0.02, 0.04) | -0.01 (-0.02, 0.00) | -0.02 (-0.04, 0.00) |
|  | TyG-WHtR | -1.33 (-3.04, 0.38) | -1.41 (-6.21, 3.39) | -1.56 (-3.38, 0.25) | -3.23 (-6.87, 0.42) |
|  | TyG-WWI | -0.06 (-0.14, 0.02) | -0.06 (-0.29, 0.17) | -0.07 (-0.16, 0.01) | -0.15 (-0.33, 0.03) |
|  | TyG-ABSI | -0.76 (-1.89, 0.37) | 0.38 (-2.80, 3.56) | -1.06 (-2.25, 0.14) | -2.13 (-4.54, 0.27) |
| **BMI** |  |  |  |  |  |
| Non overweight | TyG | -0.26 (-0.78, 0.26) | 0.81 (-1.22, 2.85) | -0.62 (-1.41, 0.17) | -0.43 (-1.99, 1.12) |
|  | TyG-BMI | -0.00 (-0.02, 0.01) | 0.03 (-0.03, 0.09) | -0.01 (-0.04, 0.02) | -0.01 (-0.06, 0.04) |
|  | TyG-WC | -0.00 (-0.01, 0.00) | 0.00 (-0.02, 0.02) | -0.01 (-0.02, -0.00)* | -0.01 (-0.03, 0.00) |
|  | TyG-WHtR | -0.69 (-1.45, 0.07) | -0.32 (-3.28, 2.64) | -1.42 (-2.58, -0.26) | -1.88 (-4.14, 0.39) |
|  | TyG-WWI | -0.03 (-0.07, 0.00) | -0.02 (-0.16, 0.12) | -0.07 (-0.12, -0.01)* | -0.09 (-0.19, 0.02) |
|  | TyG-ABSI | -0.45 (-0.92, 0.02) | -0.16 (-1.98, 1.66) | -0.91 (-1.62, -0.20)* | -1.18 (-2.56, 0.22) |
| Overweight | TyG | -0.17 (-0.65, 0.32) | -0.57 (-2.28, 1.14) | -0.17 (-0.97, 0.63) | -0.59 (-2.08, 0.90) |
|  | TyG-BMI | -0.00 (-0.02, 0.01) | -0.01 (-0.06, 0.04) | -0.01 (-0.03, 0.02) | -0.02 (-0.06, 0.03) |
|  | TyG-WC | -0.00 (-0.01, 0.00) | -0.00 (-0.02, 0.01) | -0.00 (-0.01, 0.01) | -0.00 (-0.02, 0.01) |
|  | TyG-WHtR | -0.41 (-1.06, 0.25) | -2.00 (-4.28, 0.29) | -0.31 (-1.39, 0.76) | -0.82 (-2.83, 1.18) |
|  | TyG-WWI | -0.02 (-0.06, 0.01) | -0.11 (-0.22, 0.01) | -0.02 (-0.07, 0.04) | -0.04 (-0.14, 0.06) |
|  | TyG-ABSI | -0.28 (-0.74, 0.18) | -1.01 (-2.62, 0.60) | -0.16 (-0.91, 0.60) | -0.50 (-1.91, 0.91) |

Notes: MoCA, Montreal Cognitive Assessment; DSST, Digit Symbol Substitution Test; AVLT-3, Auditory Verbal Learning Test-Immediate Recall Trial 3; AVLT-5, Auditory Verbal Learning Test-Delayed Recall; CI, confidence interval; TyG, triglyceride-glucose index; WHtR, waist-to-height ratio; BMI, body mass index; WC, waist circumference; WWI, weight-adjusted waist index; ABSI, a body shape index.

Adjusted for gender, age, education level, alcohol consumption, smoking status, BMI, WC, total cholesterol, physical activity, and history of hypertension. To avoid over-adjustment bias, the corresponding anthropometric component was excluded from.

* p < 0.05; ** p < 0.01.
