# Supplementary material for: Association of TyG index and obesity indicators with cognitive function: a cross - sectional study from Chinese health check-up centers
Source: BMC Endocr Disord. 2026 Apr 17;26:169. doi: 10.1186/s12902-026-02280-4 (PMC13224721; doi:10.1186/s12902-026-02280-4)
Supplement: Supplementary file 9 — Supplementary Material 9 [file 12902_2026_2280_MOESM9_ESM.docx]

Table S6. Interaction effects p-value of TyG and related obesity indices with cognitive function outcomes across subgroups

|  |  | **MoCA** | | **DSST** | | **AVLT-3** | | **AVLT-5** | |
| --- | --- | --- | --- | --- | --- | --- | --- | --- | --- |
|  |  | **Model 1** | **Model 2** | **Model 1** | **Model 2** | **Model 1** | **Model 2** | **Model 1** | **Model 2** |
| **TyG** | Sex | 0.008 | 0.49 | <0.001 | 0.078 | 0.133 | 0.747 | 0.295 | 0.931 |
|  | Age Group | 0.933 | 0.483 | 0.126 | 0.169 | 0.802 | 0.478 | 0.595 | 0.305 |
|  | BMI | 0.528 | 0.645 | 0.967 | 0.841 | 0.434 | 0.47 | 0.912 | 0.83 |
| **TyG-BMI** | Sex | 0.114 | 0.74 | <0.001 | 0.006 | 0.355 | 0.913 | 0.495 | 0.922 |
|  | Age Group | 0.995 | 0.372 | 0.096 | 0.265 | 0.82 | 0.505 | 0.512 | 0.292 |
|  | BMI | 0.976 | 0.56 | 0.886 | 0.686 | 0.671 | 0.37 | 0.962 | 0.705 |
| **TyG-WC** | Sex | 0.005 | 0.189 | <0.001 | 0.002 | 0.017 | 0.144 | 0.061 | 0.334 |
|  | Age Group | 0.654 | 0.304 | 0.01 | 0.12 | 0.952 | 0.376 | 0.633 | 0.219 |
|  | BMI | 0.719 | 0.44 | 0.585 | 0.369 | 0.38 | 0.242 | 0.804 | 0.588 |
| **TyG-WHtR** | Sex | 0.002 | 0.13 | <0.001 | 0.001 | 0.033 | 0.264 | 0.056 | 0.367 |
|  | Age Group | 0.862 | 0.141 | 0.096 | 0.442 | 0.927 | 0.432 | 0.58 | 0.225 |
|  | BMI | 0.927 | 0.773 | 0.687 | 0.915 | 0.701 | 0.462 | 0.848 | 0.887 |
| **TyG-WWI** | Sex | <0.001 | 0.075 | <0.001 | 0.005 | 0.01 | 0.203 | 0.022 | 0.299 |
|  | Age Group | 0.721 | 0.131 | 0.163 | 0.523 | 0.936 | 0.431 | 0.626 | 0.24 |
|  | BMI | 0.892 | 0.918 | 0.698 | 0.638 | 0.614 | 0.585 | 0.846 | 0.876 |
| **TyG-ABSI** | Sex | <0.001 | 0.098 | <0.001 | 0.019 | 0.006 | 0.138 | 0.025 | 0.298 |
|  | Age Group | 0.902 | 0.279 | 0.044 | 0.221 | 0.979 | 0.41 | 0.72 | 0.264 |
|  | BMI | 0.62 | 0.767 | 0.814 | 0.965 | 0.386 | 0.456 | 0.935 | 0.982 |

Notes: MoCA, Montreal Cognitive Assessment; DSST, Digit Symbol Substitution Test; AVLT-3, Auditory Verbal Learning Test-Immediate Recall Trial 3; AVLT-5, Auditory Verbal Learning Test-Delayed Recall; CI, confidence interval; TyG, triglyceride-glucose index; WHtR, waist-to-height ratio; BMI, body mass index; WC, waist circumference; WWI, weight-adjusted waist index; ABSI, a body shape index.

Model 1 Adjusted for gender and age

Model 2 Adjusted for gender, age, education level, alcohol consumption, smoking status, BMI, WC, total cholesterol, physical activity, and history of hypertension. To avoid over-adjustment bias, the corresponding anthropometric component was excluded from covariates in models for each composite index.
