# Supplementary material for: Association of TyG index and obesity indicators with cognitive function: a cross - sectional study from Chinese health check-up centers
Source: BMC Endocr Disord. 2026 Apr 17;26:169. doi: 10.1186/s12902-026-02280-4 (PMC13224721; doi:10.1186/s12902-026-02280-4)
Supplement: Supplementary file 10 — Supplementary Material 10 [file 12902_2026_2280_MOESM10_ESM.docx]

Table S7. Age quartile subgroup analyses of the association between TyG and related obesity indices with cognitive function (Model1)

| **Age Group** | **Exposure** | **MoCA**  **Beta (95%CI)** | **DSST**  **Beta (95%CI)** | **AVLT-3**  **Beta (95%CI)** | **AVLT-5**  **Beta (95%CI)** |
| --- | --- | --- | --- | --- | --- |
| Quartile 1 | TyG | 0.07 (-0.41, 0.55) | -1.80 (-4.42, 0.82) | 0.43 (-0.62, 1.47) | 1.22 (-0.76, 3.19) |
|  | TyG-BMI | 0.00 (-0.01, 0.01) | -0.01 (-0.06, 0.03) | 0.01 (-0.01, 0.03) | 0.03 (-0.01, 0.06) |
|  | TyG-WC | -0.00 (-0.00, 0.00) | -0.01 (-0.02, 0.01) | 0.00 (-0.00, 0.01) | 0.01 (-0.00, 0.02) |
|  | TyG-WHtR | 0.14 (-0.34, 0.61) | -1.62 (-4.22, 0.98) | 0.36 (-0.67, 1.40) | 1.34 (-0.62, 3.30) |
|  | TyG-WWI | 0.01 (-0.03, 0.04) | -0.17 (-0.36, 0.01) | 0.02 (-0.06, 0.09) | 0.06 (-0.08, 0.20) |
|  | TyG-ABSI | -0.02 (-0.50, 0.46) | -2.23 (-4.85, 0.40) | 0.23 (-0.82, 1.28) | 0.65 (-1.33, 2.62) |
| Quartile 2 | TyG | -0.34 (-0.91, 0.23) | -0.59 (-3.20, 2.03) | -0.43 (-1.51, 0.65) | -0.91 (-2.99, 1.17) |
|  | TyG-BMI | -0.01 (-0.02, 0.00) | -0.01 (-0.06, 0.04) | -0.01 (-0.03, 0.01) | -0.03 (-0.07, 0.01) |
|  | TyG-WC | -0.00 (-0.01, 0.00) | -0.00 (-0.02, 0.01) | -0.00 (-0.01, 0.00) | -0.01 (-0.02, 0.01) |
|  | TyG-WHtR | -0.54 (-1.14, 0.06) | -0.74 (-3.44, 1.96) | -0.47 (-1.61, 0.67) | -1.18 (-3.36, 1.00) |
|  | TyG-WWI | -0.03 (-0.07, 0.01) | -0.05 (-0.23, 0.13) | -0.02 (-0.10, 0.06) | -0.04 (-0.19, 0.10) |
|  | TyG-ABSI | -0.30 (-0.87, 0.27) | -0.39 (-2.98, 2.20) | -0.22 (-1.30, 0.87) | -0.36 (-2.43, 2.04) |
| Quartile3 | TyG | -0.41 (-1.16, 0.34) | -0.50 (-2.89, 1.89) | -0.87 (-1.91, 0.18) | -1.32 (-3.32, 0.67) |
|  | TyG-BMI | -0.01 (-0.02, 0.01) | -0.04 (-0.09, 0.01) | -0.02 (-0.04, 0.00) | -0.03 (-0.07, 0.01) |
|  | TyG-WC | -0.00 (-0.01, 0.00) | -0.01 (-0.02, 0.01) | -0.01 (-0.01, 0.00) | -0.01 (-0.02, 0.01) |
|  | TyG-WHtR | -0.52 (-1.30, 0.26) | -1.68 (-4.17, 0.81) | -1.20 (-2.29, -0.11) | -1.96 (-4.02, 0.10) |
|  | TyG-WWI | -0.04 (-0.09, 0.01) | -0.07 (-0.23, 0.09) | -0.08 (-0.15, -0.01) | -0.13 (-0.26, 0.00) |
|  | TyG-ABSI | -0.46 (-1.15, 0.23) | -0.37 (-2.59, 1.85) | -0.88 (-1.85, 0.09) | -1.46 (-3.29, 0.38) |
| Quartile 4 | TyG | -0.37 (-1.40, 0.67) | 2.10 (-0.79, 4.99) | -0.39 (-1.45, 0.68) | -0.28 (-2.35, 1.80) |
|  | TyG-BMI | -0.01 (-0.03, 0.01) | 0.01 (-0.04, 0.06) | -0.01 (-0.03, 0.01) | -0.02 (-0.06, 0.02) |
|  | TyG-WC | -0.00 (-0.01, 0.00) | 0.00 (-0.01, 0.02) | -0.00 (-0.01, 0.00) | -0.01 (-0.02, 0.01) |
|  | TyG-WHtR | -0.69 (-1.60, 0.22) | -0.12 (-2.65, 2.42) | -0.59 (-1.53, 0.35) | -1.09 (-2.93, 0.74) |
|  | TyG-WWI | -0.05 (-0.11, 0.01) | -0.00 (-0.18, 0.18) | -0.04 (-0.11, 0.03) | -0.06 (-0.19, 0.07) |
|  | TyG-ABSI | -0.60 (-1.54, 0.34) | 0.73 (-1.89, 3.35) | -0.56 (-1.53, 0.41) | -0.62 (-2.53, 1.30) |

Notes: MoCA, Montreal Cognitive Assessment; DSST, Digit Symbol Substitution Test; AVLT-3, Auditory Verbal Learning Test-Immediate Recall Trial 3; AVLT-5, Auditory Verbal Learning Test-Delayed Recall; CI, confidence interval; TyG, triglyceride-glucose index; WHtR, waist-to-height ratio; BMI, body mass index; WC, waist circumference; WWI, weight-adjusted waist index; ABSI, a body shape index.

Adjusted for gender and age.

* p < 0.05; ** p < 0.01.
