# Supplementary material for: Association of TyG index and obesity indicators with cognitive function: a cross - sectional study from Chinese health check-up centers
Source: BMC Endocr Disord. 2026 Apr 17;26:169. doi: 10.1186/s12902-026-02280-4 (PMC13224721; doi:10.1186/s12902-026-02280-4)
Supplement: Supplementary file 11 — Supplementary Material 11 [file 12902_2026_2280_MOESM11_ESM.docx]

Table S8. Age quartile subgroup analyses of the association between TyG and related obesity indices with cognitive function (Model2)

| **Age Group** | **Exposure** | **MoCA**  **Beta (95%CI)** | **DSST**  **Beta (95%CI)** | **AVLT-3**  **Beta (95%CI)** | **AVLT-5**  **Beta (95%CI)** |
| --- | --- | --- | --- | --- | --- |
| Quartile 1 | TyG | 0.09 (-0.45, 0.63) | -1.85 (-4.61, 0.91) | 0.36 (-0.83, 1.54) | 0.81 (-1.40, 3.02) |
|  | TyG-BMI | 0.01 (-0.00, 0.02) | -0.01 (-0.08, 0.06) | 0.01 (-0.02, 0.04) | 0.04 (-0.02, 0.10) |
|  | TyG-WC | -0.00 (-0.01, 0.00) | -0.01 (-0.04, 0.01) | 0.00 (-0.01, 0.01) | 0.00 (-0.02, 0.02) |
|  | TyG-WHtR | 0.09 (-0.68, 0.85) | -3.40 (-7.29, 0.49) | 0.17 (-1.51, 1.84) | 0.58 (-2.52, 3.67) |
|  | TyG-WWI | 0.01 (-0.03, 0.04) | -0.18 (-0.37, 0.02) | 0.01 (-0.08, 0.09) | 0.03 (-0.12, 0.19) |
|  | TyG-ABSI | -0.03 (-0.52, 0.47) | -2.08 (-4.63, 0.47) | 0.16 (-0.94, 1.26) | 0.31 (-1.72, 2.34) |
| Quartile 2 | TyG | 0.01 (-0.60, 0.61) | -0.64 (-3.48, 2.21) | -0.20 (-1.42, 1.02) | -0.61 (-2.94, 1.72) |
|  | TyG-BMI | -0.00 (-0.02, 0.01) | -0.01 (-0.09, 0.06) | -0.01 (-0.04, 0.02) | -0.03 (-0.09, 0.03) |
|  | TyG-WC | -0.00 (-0.01, 0.00) | -0.00 (-0.03, 0.02) | 0.00 (-0.01, 0.01) | -0.00 (-0.02, 0.02) |
|  | TyG-WHtR | -0.07 (-0.91, 0.78) | -1.12 (-5.05, 2.82) | 0.11 (-1.60, 1.81) | -0.14 (-3.38, 3.10) |
|  | TyG-WWI | -0.00 (-0.04, 0.04) | -0.05 (-0.25, 0.14) | 0.01 (-0.08, 0.09) | -0.01 (-0.17, 0.15) |
|  | TyG-ABSI | -0.06 (-0.63, 0.51) | -0.55 (-3.21, 2.12) | 0.03 (-1.12, 1.18) | -0.16 (-2.36, 2.04) |
| Quartile3 | TyG | -0.12 (-0.78, 0.54) | 0.64 (-1.36, 2.63) | -0.52 (-1.57, 0.52) | -0.66 (-2.68, 1.36) |
|  | TyG-BMI | 0.01 (-0.02, 0.03) | -0.01 (-0.07, 0.06) | -0.01 (-0.04, 0.02) | -0.01 (-0.07, 0.05) |
|  | TyG-WC | -0.00 (-0.01, 0.00) | 0.01 (-0.01, 0.03) | -0.00 (-0.01, 0.01) | -0.01 (-0.02, 0.01) |
|  | TyG-WHtR | -0.27 (-1.17, 0.63) | 1.01 (-1.73, 3.74) | -0.95 (-2.37, 0.48) | -1.45 (-4.19, 1.29) |
|  | TyG-WWI | -0.02 (-0.06, 0.03) | 0.05 (-0.08, 0.19) | -0.05 (-0.12, 0.02) | -0.08 (-0.21, 0.06) |
|  | TyG-ABSI | -0.19 (-0.78, 0.40) | 0.86 (-0.94, 2.65) | -0.58 (-1.51, 0.36) | -0.88 (-2.68, 0.93) |
| Quartile 4 | TyG | -0.32 (-1.32, 0.69) | 2.74 (-0.21, 5.68) | -0.74 (-1.93, 0.45) | -0.77 (-3.09, 1.54) |
|  | TyG-BMI | 0.00 (-0.03, 0.03) | 0.05 (-0.03, 0.12) | -0.00 (-0.03, 0.03) | -0.01 (-0.07, 0.05) |
|  | TyG-WC | -0.01 (-0.02, 0.00) | 0.01 (-0.01, 0.04) | -0.01 (-0.02, 0.00) | -0.01 (-0.03, 0.01) |
|  | TyG-WHtR | -1.22 (-2.56, 0.13) | -0.16 (-4.11, 3.80) | -1.29 (-2.89, 0.32) | -1.71 (-4.86, 1.44) |
|  | TyG-WWI | -0.06 (-0.12, 0.01) | -0.00 (-0.20, 0.19) | -0.07 (-0.14, 0.01) | -0.09 (-0.24, 0.07) |
|  | TyG-ABSI | -0.74 (-1.62, 0.15) | 0.66 (-1.94, 3.25) | -0.95 (-2.00, 0.10) | -1.19 (-3.26, 0.89) |

Notes: MoCA, Montreal Cognitive Assessment; DSST, Digit Symbol Substitution Test; AVLT-3, Auditory Verbal Learning Test-Immediate Recall Trial 3; AVLT-5, Auditory Verbal Learning Test-Delayed Recall; CI, confidence interval; TyG, triglyceride-glucose index; WHtR, waist-to-height ratio; BMI, body mass index; WC, waist circumference; WWI, weight-adjusted waist index; ABSI, a body shape index.

Adjusted for gender, age, education level, alcohol consumption, smoking status, BMI, WC, total cholesterol, physical activity, and history of hypertension. To avoid over-adjustment bias, the corresponding anthropometric component was excluded from covariates in models for each composite index.

* p < 0.05; ** p < 0.01.
