# Supplementary material for: Association of TyG index and obesity indicators with cognitive function: a cross - sectional study from Chinese health check-up centers
Source: BMC Endocr Disord. 2026 Apr 17;26:169. doi: 10.1186/s12902-026-02280-4 (PMC13224721; doi:10.1186/s12902-026-02280-4)
Supplement: Supplementary file 12 — Supplementary Material 12 [file 12902_2026_2280_MOESM12_ESM.docx]

| **Outcome** | **Exposure** | **Sample Size** | **Model 1** | | **Model 2** | |
| --- | --- | --- | --- | --- | --- | --- |
|  |  |  | **Beta (95%CI)** | ***P* value** | **Beta (95%CI)** | ***P* value** |
| **MoCA** | TyG | 663 | -0.31 (-0.69, 0.07) | 0.168 | -0.12 (-0.50, 0.25) | 0.699 |
|  | TyG-BMI |  | -0.01 (-0.01, 0.00) | 0.088 | 0.00 (-0.01, 0.01) | 0.854 |
|  | TyG-WC |  | -0.00 (-0.01, -0.00) | 0.038 | -0.00 (-0.01, 0.00) | 0.293 |
|  | TyG-WHtR |  | -0.56 (-0.93, -0.19) | 0.021 | -0.55 (-1.06, -0.03) | 0.293 |
|  | TyG-WWI |  | -0.04 (-0.07, -0.01) | 0.020 | -0.03 (-0.05, -0.00) | 0.293 |
|  | TyG-ABSI |  | -0.51 (-0.87, -0.14) | 0.033 | -0.36 (-0.70, -0.02) | 0.293 |
| **DSST** | TyG | 657 | -0.60 (-2.04, 0.83) | 0.470 | 0.22 (-1.21, 1.65) | 0.913 |
|  | TyG-BMI |  | -0.01 (-0.04, 0.01) | 0.339 | 0.01 (-0.02, 0.05) | 0.635 |
|  | TyG-WC |  | -0.01 (-0.01, 0.00) | 0.247 | -0.00 (-0.01, 0.01) | 0.925 |
|  | TyG-WHtR |  | -1.54 (-2.92, -0.15) | 0.072 | -1.29 (-3.25, 0.66) | 0.418 |
|  | TyG-WWI |  | -0.12 (-0.21, -0.02) | 0.048 | -0.07 (-0.16, 0.03) | 0.418 |
|  | TyG-ABSI |  | -1.33 (-2.71, 0.05) | 0.110 | -0.56 (-1.86, 0.74) | 0.562 |
| **AVLT-3** | TyG | 657 | -0.42 (-0.97, 0.13) | 0.197 | -0.39 (-0.98, 0.21) | 0.418 |
|  | TyG-BMI |  | -0.01 (-0.02, 0.00) | 0.214 | 0.00 (-0.02, 0.02) | 0.925 |
|  | TyG-WC |  | -0.00 (-0.01, 0.00) | 0.077 | -0.01 (-0.01, -0.00) | 0.293 |
|  | TyG-WHtR |  | -0.64 (-1.17, -0.11) | 0.055 | -0.98 (-1.80, -0.16) | 0.293 |
|  | TyG-WWI |  | -0.05 (-0.09, -0.01) | 0.038 | -0.05 (-0.09, -0.01) | 0.293 |
|  | TyG-ABSI |  | -0.68 (-1.20, -0.15) | 0.043 | -0.66 (-1.21, -0.12) | 0.293 |
| **AVLT-5** | TyG | 637 | -0.60 (-1.65, 0.45) | 0.321 | -0.54 (-1.69, 0.60) | 0.538 |
|  | TyG-BMI |  | -0.01 (-0.03, 0.01) | 0.303 | 0.01 (-0.03, 0.04) | 0.887 |
|  | TyG-WC |  | -0.01 (-0.01, 0.00) | 0.115 | -0.01 (-0.02, 0.00) | 0.293 |
|  | TyG-WHtR |  | -1.07 (-2.09, -0.05) | 0.084 | -1.67 (-3.24, -0.10) | 0.293 |
|  | TyG-WWI |  | -0.08 (-0.15, -0.01) | 0.061 | -0.08 (-0.16, -0.00) | 0.293 |
|  | TyG-ABSI |  | -1.09 (-2.10, -0.08) | 0.077 | -1.08 (-2.13, -0.04) | 0.293 |

Table S9. Association of TyG and related obesity indices with cognitive function (Excluding drug use,and history of diabetes and cardiovascular disease)

Notes: MCI, Mild Cognitive Impairment; CI, confidence interval; OR, odds ratio; TyG, triglyceride-glucose index; WHtR, waist-to-height ratio; BMI, body mass index; WC, waist circumference; WWI, weight-adjusted waist index; ABSI, a body shape index.

Model 1 Adjusted for gender and age

Model 2 Adjusted for gender, age, education level, alcohol consumption, smoking status, BMI, WC, total cholesterol, physical activity, and history of hypertension. The 95% CIs are unadjusted; p-values are FDR-adjusted using the Benjamini–Hochberg procedure. To avoid over-adjustment bias, the corresponding anthropometric component was excluded from covariates in models for each composite index.
