# Supplementary material for: Association of TyG index and obesity indicators with cognitive function: a cross - sectional study from Chinese health check-up centers
Source: BMC Endocr Disord. 2026 Apr 17;26:169. doi: 10.1186/s12902-026-02280-4 (PMC13224721; doi:10.1186/s12902-026-02280-4)
Supplement: Supplementary file 13 — Supplementary Material 13 [file 12902_2026_2280_MOESM13_ESM.docx]

Table S10. Association of TyG and related obesity indices with MoCA after missing value imputation.

|  | **Model 1** | | **Model 2** | |
| --- | --- | --- | --- | --- |
|  | **OR (95%CI)** | ***P* value** | **OR (95%CI)** | ***P* value** |
| TyG | 0.68 (0.48, 0.97) | 0.041 | 0.86 (0.54, 1.38) | 0.535 |
| TyG-BMI | 0.99 (0.99, 1.00) | 0.022 | 1.00 (0.98, 1.01) | 0.535 |
| TyG-WC | 1.00 (1.00, 1.00) | 0.008 | 1.00 (0.99, 1.00) | 0.485 |
| TyG-WHtR | 0.56 (0.39, 0.80) | 0.004 | 0.73 (0.38, 1.37) | 0.485 |
| TyG-WWI | 0.96 (0.94, 0.98) | 0.004 | 0.98 (0.95, 1.01) | 0.485 |
| TyG-ABSI | 0.80 (0.52, 1.22) | 0.297 | 0.80 (0.52, 1.22) | 0.485 |

Notes: MCI, Mild Cognitive Impairment; CI, confidence interval; OR, odds ratio; TyG, triglyceride-glucose index; WHtR, waist-to-height ratio; BMI, body mass index; WC, waist circumference; WWI, weight-adjusted waist index; ABSI, a body shape index.

Model 1 Adjusted for gender and age

Model 2 Adjusted for gender, age, education level, alcohol consumption, smoking status, BMI, WC, total cholesterol, physical activity, and history of hypertension. To avoid over-adjustment bias, the corresponding anthropometric component was excluded from covariates in models for each composite index.
