# Supplementary material for: Association of TyG index and obesity indicators with cognitive function: a cross - sectional study from Chinese health check-up centers
Source: BMC Endocr Disord. 2026 Apr 17;26:169. doi: 10.1186/s12902-026-02280-4 (PMC13224721; doi:10.1186/s12902-026-02280-4)
Supplement: Supplementary file 14 — Supplementary Material 14 [file 12902_2026_2280_MOESM14_ESM.docx]

Table S11. Independent and joint effects of the TyG index and obesity-related indices.

| **Outcome** |  | **Model A** | **Model B** | **Model C** |
| --- | --- | --- | --- | --- |
|  |  | **Beta (95%CI)** | **Beta (95%CI)** | **Beta (95%CI)** |
| **MoCA** | TyG | -0.27 (-0.60, 0.06) | - | -0.22 (-0.57, 0.13) |
|  | BMI | - | -0.04 (-0.11, 0.02) | -0.03 (-0.10, 0.04) |
| **DSST** | TyG | -0.02 (-1.25, 1.21) | - | 0.03 (-1.27, 1.33) |
|  | BMI | - | -0.03 (-0.28, 0.22) | -0.03 (-0.29, 0.23) |
| **AVLT-3** | TyG | -0.37 (-0.89, 0.15) | - | -0.36 (-0.91, 0.19) |
|  | BMI | - | -0.03 (-0.13, 0.07) | -0.01 (-0.12, 0.10) |
| **AVLT-5** | TyG | -0.58 (-1.58, 0.41) | - | -0.52 (-1.57, 0.53) |
|  | BMI | - | -0.07 (-0.27, 0.13) | -0.04 (-0.25, 0.17) |
| **MoCA** | TyG | -0.27 (-0.60, 0.06) | - | -0.17 (-0.52, 0.19) |
|  | WC | - | **-0.03 (-0.05, -0.00)*** | -0.02 (-0.05, 0.00) |
| **DSST** | TyG | -0.02 (-1.25, 1.21) | - | 0.10 (-1.20, 1.41) |
|  | WC | - | -0.03 (-0.11, 0.06) | -0.03 (-0.12, 0.07) |
| **AVLT-3** | TyG | -0.37 (-0.89, 0.15) | - | -0.31 (-0.86, 0.24) |
|  | WC | - | -0.02 (-0.06, 0.02) | -0.01 (-0.05, 0.03) |
| **AVLT-5** | TyG | -0.58 (-1.58, 0.41) | - | -0.41 (-1.46, 0.65) |
|  | WC | - | -0.05 (-0.12, 0.02) | -0.04 (-0.12, 0.04) |
| **MoCA** | TyG | -0.27 (-0.60, 0.06) | - | -0.16 (-0.52, 0.19) |
|  | WHtR | - | **-4.43 (-8.38, -0.47)*** | -3.77 (-7.96, 0.43) |
| **DSST** | TyG | -0.02 (-1.25, 1.21) | - | 0.34 (-0.97, 1.65) |
|  | WHtR | - | -11.25 (-25.89, 3.40) | -12.65 (-28.24, 2.95) |
| **AVLT-3** | TyG | -0.37 (-0.89, 0.15) | - | -0.28 (-0.84, 0.27) |
|  | WHtR | - | -4.19 (-10.39, 2.01) | -3.03 (-9.63, 3.57) |
| **AVLT-5** | TyG | -0.58 (-1.58, 0.41) | - | -0.38 (-1.44, 0.68) |
|  | WHtR | - | -8.65 (-20.58, 3.28) | -7.09 (-19.80, 5.63) |
| **MoCA** | TyG | -0.27 (-0.60, 0.06) | - | -0.16 (-0.51, 0.19) |
|  | WWI | - | **-0.36 (-0.67, -0.04)*** | -0.31 (-0.64, 0.03) |
| **DSST** | TyG | -0.02 (-1.25, 1.21) | - | 0.23 (-1.08, 1.54) |
|  | WWI | - | -0.62 (-1.77, 0.54) | -0.69 (-1.92, 0.54) |
| **AVLT-3** | TyG | -0.37 (-0.89, 0.15) | - | -0.29 (-0.85, 0.26) |
|  | WWI | - | -0.31 (-0.80, 0.18) | -0.21 (-0.74, 0.31) |
| **AVLT-5** | TyG | -0.58 (-1.58, 0.41) | - | -0.39 (-1.45, 0.67) |
|  | WWI | - | -0.66 (-1.61, 0.28) | -0.54 (-1.54, 0.46) |
| **MoCA** | TyG | -0.27 (-0.60, 0.06) | - | -0.24 (-0.57, 0.10) |
|  | ABSI | - | **-5.01 (-9.85, -0.16)*** | -4.62 (-9.49, 0.25) |
| **DSST** | TyG | -0.02 (-1.25, 1.21) | - | 0.10 (-1.14, 1.34) |
|  | ABSI | - | -14.97 (-32.85, 2.92) | -15.13 (-33.15, 2.89) |
| **AVLT-3** | TyG | -0.37 (-0.89, 0.15) | - | -0.33 (-0.86, 0.19) |
|  | ABSI | - | -5.23 (-12.85, 2.39) | -4.68 (-12.35, 2.99) |
| **AVLT-5** | TyG | -0.58 (-1.58, 0.41) | - | -0.51 (-1.51, 0.49) |
|  | ABSI | - | -9.87 (-24.49, 4.75) | -8.99 (-23.71, 5.74) |

Notes: MCI, Mild Cognitive Impairment; CI, confidence interval; OR, odds ratio; TyG, triglyceride-glucose index; WHtR, waist-to-height ratio; BMI, body mass index; WC, waist circumference; WWI, weight-adjusted waist index; ABSI, a body shape index.

Model 1 Adjusted for gender and age

Model 2 Adjusted for gender, age, education level, alcohol consumption, smoking status, BMI, WC, total cholesterol, physical activity, and history of hypertension. To avoid over-adjustment bias, the corresponding anthropometric component was excluded from covariates in models for each composite index.

* p < 0.05; ** p < 0.01.
