# Supplementary material for: Association of TyG index and obesity indicators with cognitive function: a cross - sectional study from Chinese health check-up centers
Source: BMC Endocr Disord. 2026 Apr 17;26:169. doi: 10.1186/s12902-026-02280-4 (PMC13224721; doi:10.1186/s12902-026-02280-4)
Supplement: Supplementary file 15 — Supplementary Material 15 [file 12902_2026_2280_MOESM15_ESM.docx]

Table S12. Association of TyG and related obesity indices with MCI.

|  | **Model 1** | | **Model 2** | |
| --- | --- | --- | --- | --- |
|  | **OR (95%CI)** | ***P* value** | **OR (95%CI)** | ***P* value** |
| TyG | 1.01 (0.80, 1.27) | 0.968 | 0.93 (0.70, 1.23) | 0.592 |
| TyG-BMI | 1.00 (1.00, 1.01) | 0.692 | 1.00 (0.99, 1.01) | 0.618 |
| TyG-WC | 1.00 (1.00, 1.00) | 0.693 | 1.00 (1.00, 1.00) | 0.616 |
| TyG-WHtR | 1.04 (0.82, 1.32) | 0.725 | 0.86 (0.58, 1.28) | 0.464 |
| TyG-WWI | 1.00 (0.99, 1.02) | 0.905 | 0.99 (0.97, 1.01) | 0.425 |
| TyG-ABSI | 1.00 (0.80, 1.26) | 0.973 | 0.91 (0.70, 1.18) | 0.477 |

Notes: MCI, Mild Cognitive Impairment; CI, confidence interval; OR, odds ratio; TyG, triglyceride-glucose index; WHtR, waist-to-height ratio; BMI, body mass index; WC, waist circumference; WWI, weight-adjusted waist index; ABSI, a body shape index.

Model 1 Adjusted for gender and age

Model 2 Adjusted for gender, age, education level, alcohol consumption, smoking status, BMI, WC, total cholesterol, physical activity, and history of hypertension. The 95% CIs are unadjusted; p-values are FDR-adjusted using the Benjamini–Hochberg procedure. To avoid over-adjustment bias, the corresponding anthropometric component was excluded from covariates in models for each composite index.
