# Supplementary material for: Association of TyG index and obesity indicators with cognitive function: a cross - sectional study from Chinese health check-up centers
Source: BMC Endocr Disord. 2026 Apr 17;26:169. doi: 10.1186/s12902-026-02280-4 (PMC13224721; doi:10.1186/s12902-026-02280-4)
Supplement: Supplementary file 16 — Supplementary Material 16 [file 12902_2026_2280_MOESM16_ESM.docx]

### Table S13. Predictive Value Comparison for each exposure model.

| **Outcome** | **Exposure** | **Model 1** | | | **Model 2** | | |
| --- | --- | --- | --- | --- | --- | --- | --- |
|  |  | **Adjust R^2^** | **AIC** | **BIC** | **Adjust R^2^** | **AIC** | **BIC** |
| **MoCA** | TyG | 0.219 | 3923.38 | 3946.46 | 0.416 | 3714.98 | 3779.62 |
|  | TyG-BMI | 0.220 | 3922.8 | 3945.89 | 0.415 | 3715.09 | 3775.12 |
|  | TyG-WC | 0.223 | 3919.75 | 3942.83 | 0.417 | 3713.63 | 3773.66 |
|  | TyG-WHtR | 0.225 | 3917.91 | 3941 | 0.416 | 3713.74 | 3773.77 |
|  | TyG-WWI | 0.226 | 3917.1 | 3940.19 | 0.417 | 3713.66 | 3773.69 |
|  | TyG-ABSI | 0.224 | 3919.27 | 3942.36 | 0.417 | 3713.53 | 3773.56 |
| **DSST** | TyG | 0.541 | 5807.38 | 5830.42 | 0.649 | 5616.16 | 5680.67 |
|  | TyG-BMI | 0.541 | 5806.39 | 5829.43 | 0.650 | 5614.16 | 5674.06 |
|  | TyG-WC | 0.542 | 5806.10 | 5829.14 | 0.650 | 5614.53 | 5674.43 |
|  | TyG-WHtR | 0.544 | 5802.45 | 5825.49 | 0.651 | 5612.78 | 5672.69 |
|  | TyG-WWI | 0.540 | 5801.71 | 5824.75 | 0.651 | 5612.74 | 5672.64 |
|  | TyG-ABSI | 0.542 | 5804.78 | 5827.82 | 0.650 | 5613.78 | 5673.69 |
| **AVLT-3** | TyG | 0.289 | 4401.92 | 4424.95 | 0.359 | 4333.61 | 4398.10 |
|  | TyG-BMI | 0.288 | 4402.11 | 4425.15 | 0.358 | 4333.33 | 4393.21 |
|  | TyG-WC | 0.290 | 4400.84 | 4423.87 | 0.360 | 4331.73 | 4391.62 |
|  | TyG-WHtR | 0.291 | 4399.35 | 4422.39 | 0.361 | 4330.84 | 4390.72 |
|  | TyG-WWI | 0.292 | 4398.47 | 4421.51 | 0.361 | 4330.62 | 4390.51 |
|  | TyG-ABSI | 0.291 | 4399.79 | 4422.83 | 0.361 | 4330.93 | 4390.82 |
| **AVLT-5** | TyG | 0.282 | 5198.22 | 5221.11 | 0.351 | 5134.54 | 5198.63 |
|  | TyG-BMI | 0.282 | 5197.71 | 5220.60 | 0.350 | 5133.71 | 5193.22 |
|  | TyG-WC | 0.283 | 5196.49 | 5219.38 | 0.351 | 5132.74 | 5192.25 |
|  | TyG-WHtR | 0.284 | 5195.42 | 5218.31 | 0.352 | 5132.26 | 5191.78 |
|  | TyG-WWI | 0.285 | 5195.18 | 5218.07 | 0.352 | 5132.32 | 5191.83 |
|  | TyG-ABSI | 0.283 | 5196.32 | 5219.21 | 0.351 | 5132.55 | 5192.06 |

Notes: MoCA, Montreal Cognitive Assessment; DSST, Digit Symbol Substitution Test; AVLT-3, Auditory Verbal Learning Test-Immediate Recall Trial 3; AVLT-5, Auditory Verbal Learning Test-Delayed Recall; CI, confidence interval; TyG, triglyceride-glucose index; WHtR, waist-to-height ratio; BMI, body mass index; WC, waist circumference; WWI, weight-adjusted waist index; ABSI, a body shape index.

Model 1 Adjusted for gender and age

Model 2 Adjusted for gender, age, education level, alcohol consumption, smoking status, BMI, WC, total cholesterol, physical activity, and history of hypertension. To avoid over-adjustment bias, the corresponding anthropometric component was excluded from covariates in models for each composite index.
