# Supplementary material for: Association of TyG index and obesity indicators with cognitive function: a cross - sectional study from Chinese health check-up centers
Source: BMC Endocr Disord. 2026 Apr 17;26:169. doi: 10.1186/s12902-026-02280-4 (PMC13224721; doi:10.1186/s12902-026-02280-4)
Supplement: Supplementary file 17 — Supplementary Material 17 [file 12902_2026_2280_MOESM17_ESM.docx]

### Table S14. Variance Inflation Factor (VIF) results for each exposure model

|  | **TyG Model VIF** | **TyG-BMI Model VIF** | **TyG-WC Model VIF** | **TyG-WHtR Model VIF** | **TyG-WWI Model VIF** | **TyG-ABSI Model VIF** |
| --- | --- | --- | --- | --- | --- | --- |
| TyG | 1.46 | - | - | - | - | - |
| TyG-BMI | - | 3.49 | - | - | - | - |
| TyG-WC | - | - | 3.6 | - | - | - |
| TyG-WHtR | - | - | - | 3.13 | - | - |
| TyG-WWI | - | - | - | - | 1.65 | - |
| TyG-ABSI | - | - | - | - | - | 1.49 |
| Age | 1.52 | 1.48 | 1.48 | 1.54 | 1.55 | 1.52 |
| Gender | 1.86 | 1.80 | 1.78 | 1.58 | 1.58 | 1.65 |
| BMI | 4.07 | - | 2.80 | 2.84 | 1.51 | 1.32 |
| WC | 5.16 | 4.05 | - | - | - | - |
| Education level | 1.27 | 1.27 | 1.27 | 1.27 | 1.27 | 1.27 |
| Smoking | 1.14 | 1.14 | 1.14 | 1.14 | 1.14 | 1.14 |
| Alcohol | 1.35 | 1.35 | 1.35 | 1.34 | 1.34 | 1.35 |
| Activity | 1.04 | 1.04 | 1.04 | 1.04 | 1.04 | 1.04 |
| Hypertension | 1.26 | 1.25 | 1.26 | 1.25 | 1.25 | 1.26 |
| TC | 1.13 | 1.09 | 1.09 | 1.09 | 1.09 | 1.09 |

Note: VIF, variance inflation factor; TC, total cholesterol; TyG, triglyceride-glucose index; WHtR, waist-to-height ratio; BMI, body mass index; WC, waist circumference; WWI, weight-adjusted waist index; ABSI, a body shape index.
