# Supplementary material for: Association of TyG index and obesity indicators with cognitive function: a cross - sectional study from Chinese health check-up centers
Source: BMC Endocr Disord. 2026 Apr 17;26:169. doi: 10.1186/s12902-026-02280-4 (PMC13224721; doi:10.1186/s12902-026-02280-4)
Supplement: Supplementary file 18 — Supplementary Material 18 [file 12902_2026_2280_MOESM18_ESM.docx]

### Table S15. Ceiling effect test results of cognitive outcome variables.

| **Outcome** | **Mean ± SD** | **Minimum** | **Maximum** | **Skewness** | **Kurtosis** | **Ceiling ratio (%)** |
| --- | --- | --- | --- | --- | --- | --- |
| MOCA | 25.22±3.76 | 6 | 30 | -1.5 | 3.56 | 7.22 |
| DSST | 51.14±17.90 | 0 | 90 | -0.14 | -0.47 | 1.21 |
| AVLT-N3 | 17.94±5.59 | 2 | 34 | 0.06 | -0.41 | 0 |
| AVLT-N5 | 30.46±10.56 | 2 | 58 | -0.04 | -0.41 | 0 |

Note: SD, standard deviation; MoCA, Montreal Cognitive Assessment; AVLT-3, Auditory Verbal Learning Test-Immediate Recall Trial 3; AVLT-5, Auditory Verbal Learning Test-Delayed Recall.

Ceiling ratio refers to the percentage of participants achieving the maximum score of each cognitive outcome variable. A ceiling ratio > 15% indicates a notable ceiling effect, while a ratio > 50% suggests a severe ceiling effect. A ceiling ratio of 0% indicates no participant reached the maximum score, reflecting an absence of ceiling effect.
